# Measurement of the top-quark mass in the fully hadronic decay channel from ATLAS data at $\sqrt{s}=7\mathrm{\,TeV}$

**DOI:** 10.1140/epjc/s10052-015-3373-1

**Published:** 2015-04-23

**Authors:** ATLAS Collaboration, G. Aad, B. Abbott, J. Abdallah, S. Abdel Khalek, O. Abdinov, R. Aben, B. Abi, M. Abolins, O. S. AbouZeid, H. Abramowicz, H. Abreu, R. Abreu, Y. Abulaiti, B. S. Acharya, L. Adamczyk, D. L. Adams, J. Adelman, S. Adomeit, T. Adye, T. Agatonovic-Jovin, J. A. Aguilar-Saavedra, M. Agustoni, S. P. Ahlen, F. Ahmadov, G. Aielli, H. Akerstedt, T. P. A. Åkesson, G. Akimoto, A. V. Akimov, G. L. Alberghi, J. Albert, S. Albrand, M. J. Alconada Verzini, M. Aleksa, I. N. Aleksandrov, C. Alexa, G. Alexander, G. Alexandre, T. Alexopoulos, M. Alhroob, G. Alimonti, L. Alio, J. Alison, B. M. M. Allbrooke, L. J. Allison, P. P. Allport, J. Almond, A. Aloisio, A. Alonso, F. Alonso, C. Alpigiani, A. Altheimer, B. Alvarez Gonzalez, M. G. Alviggi, K. Amako, Y. Amaral Coutinho, C. Amelung, D. Amidei, S. P. Amor Dos Santos, A. Amorim, S. Amoroso, N. Amram, G. Amundsen, C. Anastopoulos, L. S. Ancu, N. Andari, T. Andeen, C. F. Anders, G. Anders, K. J. Anderson, A. Andreazza, V. Andrei, X. S. Anduaga, S. Angelidakis, I. Angelozzi, P. Anger, A. Angerami, F. Anghinolfi, A. V. Anisenkov, N. Anjos, A. Annovi, A. Antonaki, M. Antonelli, A. Antonov, J. Antos, F. Anulli, M. Aoki, L. Aperio Bella, R. Apolle, G. Arabidze, I. Aracena, Y. Arai, J. P. Araque, A. T. H. Arce, J-F. Arguin, S. Argyropoulos, M. Arik, A. J. Armbruster, O. Arnaez, V. Arnal, H. Arnold, M. Arratia, O. Arslan, A. Artamonov, G. Artoni, S. Asai, N. Asbah, A. Ashkenazi, B. Åsman, L. Asquith, K. Assamagan, R. Astalos, M. Atkinson, N. B. Atlay, B. Auerbach, K. Augsten, M. Aurousseau, G. Avolio, G. Azuelos, Y. Azuma, M. A. Baak, A. E. Baas, C. Bacci, H. Bachacou, K. Bachas, M. Backes, M. Backhaus, J. Backus Mayes, E. Badescu, P. Bagiacchi, P. Bagnaia, Y. Bai, T. Bain, J. T. Baines, O. K. Baker, P. Balek, F. Balli, E. Banas, Sw. Banerjee, A. A. E. Bannoura, V. Bansal, H. S. Bansil, L. Barak, S. P. Baranov, E. L. Barberio, D. Barberis, M. Barbero, T. Barillari, M. Barisonzi, T. Barklow, N. Barlow, B. M. Barnett, R. M. Barnett, Z. Barnovska, A. Baroncelli, G. Barone, A. J. Barr, F. Barreiro, J. Barreiro Guimarães da Costa, R. Bartoldus, A. E. Barton, P. Bartos, V. Bartsch, A. Bassalat, A. Basye, R. L. Bates, J. R. Batley, M. Battaglia, M. Battistin, F. Bauer, H. S. Bawa, M. D. Beattie, T. Beau, P. H. Beauchemin, R. Beccherle, P. Bechtle, H. P. Beck, K. Becker, S. Becker, M. Beckingham, C. Becot, A. J. Beddall, A. Beddall, S. Bedikian, V. A. Bednyakov, C. P. Bee, L. J. Beemster, T. A. Beermann, M. Begel, K. Behr, C. Belanger-Champagne, P. J. Bell, W. H. Bell, G. Bella, L. Bellagamba, A. Bellerive, M. Bellomo, K. Belotskiy, O. Beltramello, O. Benary, D. Benchekroun, K. Bendtz, N. Benekos, Y. Benhammou, E. Benhar Noccioli, J. A. Benitez Garcia, D. P. Benjamin, J. R. Bensinger, K. Benslama, S. Bentvelsen, D. Berge, E. Bergeaas Kuutmann, N. Berger, F. Berghaus, J. Beringer, C. Bernard, P. Bernat, C. Bernius, F. U. Bernlochner, T. Berry, P. Berta, C. Bertella, G. Bertoli, F. Bertolucci, C. Bertsche, D. Bertsche, M. I. Besana, G. J. Besjes, O. Bessidskaia Bylund, M. Bessner, N. Besson, C. Betancourt, S. Bethke, W. Bhimji, R. M. Bianchi, L. Bianchini, M. Bianco, O. Biebel, S. P. Bieniek, K. Bierwagen, J. Biesiada, M. Biglietti, J. Bilbao De Mendizabal, H. Bilokon, M. Bindi, S. Binet, A. Bingul, C. Bini, C. W. Black, J. E. Black, K. M. Black, D. Blackburn, R. E. Blair, J.-B. Blanchard, T. Blazek, I. Bloch, C. Blocker, W. Blum, U. Blumenschein, G. J. Bobbink, V. S. Bobrovnikov, S. S. Bocchetta, A. Bocci, C. Bock, C. R. Boddy, M. Boehler, T. T. Boek, J. A. Bogaerts, A. G. Bogdanchikov, A. Bogouch, C. Bohm, J. Bohm, V. Boisvert, T. Bold, V. Boldea, A. S. Boldyrev, M. Bomben, M. Bona, M. Boonekamp, A. Borisov, G. Borissov, M. Borri, S. Borroni, J. Bortfeldt, V. Bortolotto, K. Bos, D. Boscherini, M. Bosman, H. Boterenbrood, J. Boudreau, J. Bouffard, E. V. Bouhova-Thacker, D. Boumediene, C. Bourdarios, N. Bousson, S. Boutouil, A. Boveia, J. Boyd, I. R. Boyko, J. Bracinik, A. Brandt, G. Brandt, O. Brandt, U. Bratzler, B. Brau, J. E. Brau, H. M. Braun, S. F. Brazzale, B. Brelier, K. Brendlinger, A. J. Brennan, R. Brenner, S. Bressler, K. Bristow, T. M. Bristow, D. Britton, F. M. Brochu, I. Brock, R. Brock, C. Bromberg, J. Bronner, G. Brooijmans, T. Brooks, W. K. Brooks, J. Brosamer, E. Brost, J. Brown, P. A. Bruckman de Renstrom, D. Bruncko, R. Bruneliere, S. Brunet, A. Bruni, G. Bruni, M. Bruschi, L. Bryngemark, T. Buanes, Q. Buat, F. Bucci, P. Buchholz, R. M. Buckingham, A. G. Buckley, S. I. Buda, I. A. Budagov, F. Buehrer, L. Bugge, M. K. Bugge, O. Bulekov, A. C. Bundock, H. Burckhart, S. Burdin, B. Burghgrave, S. Burke, I. Burmeister, E. Busato, D. Büscher, V. Büscher, P. Bussey, C. P. Buszello, B. Butler, J. M. Butler, A. I. Butt, C. M. Buttar, J. M. Butterworth, P. Butti, W. Buttinger, A. Buzatu, M. Byszewski, S. Cabrera Urbán, D. Caforio, O. Cakir, P. Calafiura, A. Calandri, G. Calderini, P. Calfayan, R. Calkins, L. P. Caloba, D. Calvet, S. Calvet, R. Camacho Toro, S. Camarda, D. Cameron, L. M. Caminada, R. Caminal Armadans, S. Campana, M. Campanelli, A. Campoverde, V. Canale, A. Canepa, M. Cano Bret, J. Cantero, R. Cantrill, T. Cao, M. D. M. Capeans Garrido, I. Caprini, M. Caprini, M. Capua, R. Caputo, R. Cardarelli, T. Carli, G. Carlino, L. Carminati, S. Caron, E. Carquin, G. D. Carrillo-Montoya, J. R. Carter, J. Carvalho, D. Casadei, M. P. Casado, M. Casolino, E. Castaneda-Miranda, A. Castelli, V. Castillo Gimenez, N. F. Castro, P. Catastini, A. Catinaccio, J. R. Catmore, A. Cattai, G. Cattani, J. Caudron, S. Caughron, V. Cavaliere, D. Cavalli, M. Cavalli-Sforza, V. Cavasinni, F. Ceradini, B. C. Cerio, K. Cerny, A. S. Cerqueira, A. Cerri, L. Cerrito, F. Cerutti, M. Cerv, A. Cervelli, S. A. Cetin, A. Chafaq, D. Chakraborty, I. Chalupkova, P. Chang, B. Chapleau, J. D. Chapman, D. Charfeddine, D. G. Charlton, C. C. Chau, C. A. Chavez Barajas, S. Cheatham, A. Chegwidden, S. Chekanov, S. V. Chekulaev, G. A. Chelkov, M. A. Chelstowska, C. Chen, H. Chen, K. Chen, L. Chen, S. Chen, X. Chen, Y. Chen, Y. Chen, H. C. Cheng, Y. Cheng, A. Cheplakov, R. Cherkaoui El Moursli, V. Chernyatin, E. Cheu, L. Chevalier, V. Chiarella, G. Chiefari, J. T. Childers, A. Chilingarov, G. Chiodini, A. S. Chisholm, R. T. Chislett, A. Chitan, M. V. Chizhov, S. Chouridou, B. K. B. Chow, D. Chromek-Burckhart, M. L. Chu, J. Chudoba, J. J. Chwastowski, L. Chytka, G. Ciapetti, A. K. Ciftci, R. Ciftci, D. Cinca, V. Cindro, A. Ciocio, P. Cirkovic, Z. H. Citron, M. Citterio, M. Ciubancan, A. Clark, P. J. Clark, R. N. Clarke, W. Cleland, J. C. Clemens, C. Clement, Y. Coadou, M. Cobal, A. Coccaro, J. Cochran, L. Coffey, J. G. Cogan, J. Coggeshall, B. Cole, S. Cole, A. P. Colijn, J. Collot, T. Colombo, G. Colon, G. Compostella, P. Conde Muiño, E. Coniavitis, M. C. Conidi, S. H. Connell, I. A. Connelly, S. M. Consonni, V. Consorti, S. Constantinescu, C. Conta, G. Conti, F. Conventi, M. Cooke, B. D. Cooper, A. M. Cooper-Sarkar, N. J. Cooper-Smith, K. Copic, T. Cornelissen, M. Corradi, F. Corriveau, A. Corso-Radu, A. Cortes-Gonzalez, G. Cortiana, G. Costa, M. J. Costa, D. Costanzo, D. Côté, G. Cottin, G. Cowan, B. E. Cox, K. Cranmer, G. Cree, S. Crépé-Renaudin, F. Crescioli, W. A. Cribbs, M. Crispin Ortuzar, M. Cristinziani, V. Croft, G. Crosetti, C.-M. Cuciuc, T. Cuhadar Donszelmann, J. Cummings, M. Curatolo, C. Cuthbert, H. Czirr, P. Czodrowski, Z. Czyczula, S. D’Auria, M. D’Onofrio, M. J. Da Cunha Sargedas De Sousa, C. Da Via, W. Dabrowski, A. Dafinca, T. Dai, O. Dale, F. Dallaire, C. Dallapiccola, M. Dam, A. C. Daniells, M. Dano Hoffmann, V. Dao, G. Darbo, S. Darmora, J. A. Dassoulas, A. Dattagupta, W. Davey, C. David, T. Davidek, E. Davies, M. Davies, O. Davignon, A. R. Davison, P. Davison, Y. Davygora, E. Dawe, I. Dawson, R. K. Daya-Ishmukhametova, K. De, R. de Asmundis, S. De Castro, S. De Cecco, N. De Groot, P. de Jong, H. De la Torre, F. De Lorenzi, L. De Nooij, D. De Pedis, A. De Salvo, U. De Sanctis, A. De Santo, J. B. De Vivie De Regie, W. J. Dearnaley, R. Debbe, C. Debenedetti, B. Dechenaux, D. V. Dedovich, I. Deigaard, J. Del Peso, T. Del Prete, F. Deliot, C. M. Delitzsch, M. Deliyergiyev, A. Dell’Acqua, L. Dell’Asta, M. Dell’Orso, M. Della Pietra, D. della Volpe, M. Delmastro, P. A. Delsart, C. Deluca, S. Demers, M. Demichev, A. Demilly, S. P. Denisov, D. Derendarz, J. E. Derkaoui, F. Derue, P. Dervan, K. Desch, C. Deterre, P. O. Deviveiros, A. Dewhurst, S. Dhaliwal, A. Di Ciaccio, L. Di Ciaccio, A. Di Domenico, C. Di Donato, A. Di Girolamo, B. Di Girolamo, A. Di Mattia, B. Di Micco, R. Di Nardo, A. Di Simone, R. Di Sipio, D. Di Valentino, F. A. Dias, M. A. Diaz, E. B. Diehl, J. Dietrich, T. A. Dietzsch, S. Diglio, A. Dimitrievska, J. Dingfelder, C. Dionisi, P. Dita, S. Dita, F. Dittus, F. Djama, T. Djobava, M. A. B. do Vale, A. Do Valle Wemans, T. K. O. Doan, D. Dobos, C. Doglioni, T. Doherty, T. Dohmae, J. Dolejsi, Z. Dolezal, B. A. Dolgoshein, M. Donadelli, S. Donati, P. Dondero, J. Donini, J. Dopke, A. Doria, M. T. Dova, A. T. Doyle, M. Dris, J. Dubbert, S. Dube, E. Dubreuil, E. Duchovni, G. Duckeck, O. A. Ducu, D. Duda, A. Dudarev, F. Dudziak, L. Duflot, L. Duguid, M. Dührssen, M. Dunford, H. Duran Yildiz, M. Düren, A. Durglishvili, M. Dwuznik, M. Dyndal, J. Ebke, W. Edson, N. C. Edwards, W. Ehrenfeld, T. Eifert, G. Eigen, K. Einsweiler, T. Ekelof, M. El Kacimi, M. Ellert, S. Elles, F. Ellinghaus, N. Ellis, J. Elmsheuser, M. Elsing, D. Emeliyanov, Y. Enari, O. C. Endner, M. Endo, R. Engelmann, J. Erdmann, A. Ereditato, D. Eriksson, G. Ernis, J. Ernst, M. Ernst, J. Ernwein, D. Errede, S. Errede, E. Ertel, M. Escalier, H. Esch, C. Escobar, B. Esposito, A. I. Etienvre, E. Etzion, H. Evans, A. Ezhilov, L. Fabbri, G. Facini, R. M. Fakhrutdinov, S. Falciano, R. J. Falla, J. Faltova, Y. Fang, M. Fanti, A. Farbin, A. Farilla, T. Farooque, S. Farrell, S. M. Farrington, P. Farthouat, F. Fassi, P. Fassnacht, D. Fassouliotis, A. Favareto, L. Fayard, P. Federic, O. L. Fedin, W. Fedorko, M. Fehling-Kaschek, S. Feigl, L. Feligioni, C. Feng, E. J. Feng, H. Feng, A. B. Fenyuk, S. Fernandez Perez, S. Ferrag, J. Ferrando, A. Ferrari, P. Ferrari, R. Ferrari, D. E. Ferreira de Lima, A. Ferrer, D. Ferrere, C. Ferretti, A. Ferretto Parodi, M. Fiascaris, F. Fiedler, A. Filipčič, M. Filipuzzi, F. Filthaut, M. Fincke-Keeler, K. D. Finelli, M. C. N. Fiolhais, L. Fiorini, A. Firan, A. Fischer, J. Fischer, W. C. Fisher, E. A. Fitzgerald, M. Flechl, I. Fleck, P. Fleischmann, S. Fleischmann, G. T. Fletcher, G. Fletcher, T. Flick, A. Floderus, L. R. Flores Castillo, A. C. Florez Bustos, M. J. Flowerdew, A. Formica, A. Forti, D. Fortin, D. Fournier, H. Fox, S. Fracchia, P. Francavilla, M. Franchini, S. Franchino, D. Francis, L. Franconi, M. Franklin, S. Franz, M. Fraternali, S. T. French, C. Friedrich, F. Friedrich, D. Froidevaux, J. A. Frost, C. Fukunaga, E. Fullana Torregrosa, B. G. Fulsom, J. Fuster, C. Gabaldon, O. Gabizon, A. Gabrielli, A. Gabrielli, S. Gadatsch, S. Gadomski, G. Gagliardi, P. Gagnon, C. Galea, B. Galhardo, E. J. Gallas, V. Gallo, B. J. Gallop, P. Gallus, G. Galster, K. K. Gan, R. P. Gandrajula, J. Gao, Y. S. Gao, F. M. Garay Walls, F. Garberson, C. García, J. E. García Navarro, M. Garcia-Sciveres, R. W. Gardner, N. Garelli, V. Garonne, C. Gatti, G. Gaudio, B. Gaur, L. Gauthier, P. Gauzzi, I. L. Gavrilenko, C. Gay, G. Gaycken, E. N. Gazis, P. Ge, Z. Gecse, C. N. P. Gee, D. A. A. Geerts, Ch. Geich-Gimbel, K. Gellerstedt, C. Gemme, A. Gemmell, M. H. Genest, S. Gentile, M. George, S. George, D. Gerbaudo, A. Gershon, H. Ghazlane, N. Ghodbane, B. Giacobbe, S. Giagu, V. Giangiobbe, P. Giannetti, F. Gianotti, B. Gibbard, S. M. Gibson, M. Gilchriese, T. P. S. Gillam, D. Gillberg, G. Gilles, D. M. Gingrich, N. Giokaris, M. P. Giordani, R. Giordano, F. M. Giorgi, F. M. Giorgi, P. F. Giraud, D. Giugni, C. Giuliani, M. Giulini, B. K. Gjelsten, S. Gkaitatzis, I. Gkialas, L. K. Gladilin, C. Glasman, J. Glatzer, P. C. F. Glaysher, A. Glazov, G. L. Glonti, M. Goblirsch-Kolb, J. R. Goddard, J. Godfrey, J. Godlewski, C. Goeringer, S. Goldfarb, T. Golling, D. Golubkov, A. Gomes, L. S. Gomez Fajardo, R. Gonçalo, J. Goncalves Pinto Firmino Da Costa, L. Gonella, S. González de la Hoz, G. Gonzalez Parra, S. Gonzalez-Sevilla, L. Goossens, P. A. Gorbounov, H. A. Gordon, I. Gorelov, B. Gorini, E. Gorini, A. Gorišek, E. Gornicki, A. T. Goshaw, C. Gössling, M. I. Gostkin, M. Gouighri, D. Goujdami, M. P. Goulette, A. G. Goussiou, C. Goy, S. Gozpinar, H. M. X. Grabas, L. Graber, I. Grabowska-Bold, P. Grafström, K-J. Grahn, J. Gramling, E. Gramstad, S. Grancagnolo, V. Grassi, V. Gratchev, H. M. Gray, E. Graziani, O. G. Grebenyuk, Z. D. Greenwood, K. Gregersen, I. M. Gregor, P. Grenier, J. Griffiths, A. A. Grillo, K. Grimm, S. Grinstein, Ph. Gris, Y. V. Grishkevich, J.-F. Grivaz, J. P. Grohs, A. Grohsjean, E. Gross, J. Grosse-Knetter, G. C. Grossi, J. Groth-Jensen, Z. J. Grout, L. Guan, F. Guescini, D. Guest, O. Gueta, C. Guicheney, E. Guido, T. Guillemin, S. Guindon, U. Gul, C. Gumpert, J. Gunther, J. Guo, S. Gupta, P. Gutierrez, N. G. Gutierrez Ortiz, C. Gutschow, N. Guttman, C. Guyot, C. Gwenlan, C. B. Gwilliam, A. Haas, C. Haber, H. K. Hadavand, N. Haddad, P. Haefner, S. Hageböck, Z. Hajduk, H. Hakobyan, M. Haleem, D. Hall, G. Halladjian, K. Hamacher, P. Hamal, K. Hamano, M. Hamer, A. Hamilton, S. Hamilton, G. N. Hamity, P. G. Hamnett, L. Han, K. Hanagaki, K. Hanawa, M. Hance, P. Hanke, R. Hanna, J. B. Hansen, J. D. Hansen, P. H. Hansen, K. Hara, A. S. Hard, T. Harenberg, F. Hariri, S. Harkusha, D. Harper, R. D. Harrington, O. M. Harris, P. F. Harrison, F. Hartjes, M. Hasegawa, S. Hasegawa, Y. Hasegawa, A. Hasib, S. Hassani, S. Haug, M. Hauschild, R. Hauser, M. Havranek, C. M. Hawkes, R. J. Hawkings, A. D. Hawkins, T. Hayashi, D. Hayden, C. P. Hays, H. S. Hayward, S. J. Haywood, S. J. Head, T. Heck, V. Hedberg, L. Heelan, S. Heim, T. Heim, B. Heinemann, L. Heinrich, J. Hejbal, L. Helary, C. Heller, M. Heller, S. Hellman, D. Hellmich, C. Helsens, J. Henderson, R. C. W. Henderson, Y. Heng, C. Hengler, A. Henrichs, A. M. Henriques Correia, S. Henrot-Versille, C. Hensel, G. H. Herbert, Y. Hernández Jiménez, R. Herrberg-Schubert, G. Herten, R. Hertenberger, L. Hervas, G. G. Hesketh, N. P. Hessey, R. Hickling, E. Higón-Rodriguez, E. Hill, J. C. Hill, K. H. Hiller, S. Hillert, S. J. Hillier, I. Hinchliffe, E. Hines, M. Hirose, D. Hirschbuehl, J. Hobbs, N. Hod, M. C. Hodgkinson, P. Hodgson, A. Hoecker, M. R. Hoeferkamp, F. Hoenig, J. Hoffman, D. Hoffmann, J. I. Hofmann, M. Hohlfeld, T. R. Holmes, T. M. Hong, L. Hooft van Huysduynen, J-Y. Hostachy, S. Hou, A. Hoummada, J. Howard, J. Howarth, M. Hrabovsky, I. Hristova, J. Hrivnac, T. Hryn’ova, C. Hsu, P. J. Hsu, S.-C. Hsu, D. Hu, X. Hu, Y. Huang, Z. Hubacek, F. Hubaut, F. Huegging, T. B. Huffman, E. W. Hughes, G. Hughes, M. Huhtinen, T. A. Hülsing, M. Hurwitz, N. Huseynov, J. Huston, J. Huth, G. Iacobucci, G. Iakovidis, I. Ibragimov, L. Iconomidou-Fayard, E. Ideal, P. Iengo, O. Igonkina, T. Iizawa, Y. Ikegami, K. Ikematsu, M. Ikeno, Y. Ilchenko, D. Iliadis, N. Ilic, Y. Inamaru, T. Ince, P. Ioannou, M. Iodice, K. Iordanidou, V. Ippolito, A. Irles Quiles, C. Isaksson, M. Ishino, M. Ishitsuka, R. Ishmukhametov, C. Issever, S. Istin, J. M. Iturbe Ponce, R. Iuppa, J. Ivarsson, W. Iwanski, H. Iwasaki, J. M. Izen, V. Izzo, B. Jackson, M. Jackson, P. Jackson, M. R. Jaekel, V. Jain, K. Jakobs, S. Jakobsen, T. Jakoubek, J. Jakubek, D. O. Jamin, D. K. Jana, E. Jansen, H. Jansen, J. Janssen, M. Janus, G. Jarlskog, N. Javadov, T. Javůrek, L. Jeanty, J. Jejelava, G.-Y. Jeng, D. Jennens, P. Jenni, J. Jentzsch, C. Jeske, S. Jézéquel, H. Ji, J. Jia, Y. Jiang, M. Jimenez Belenguer, S. Jin, A. Jinaru, O. Jinnouchi, M. D. Joergensen, K. E. Johansson, P. Johansson, K. A. Johns, K. Jon-And, G. Jones, R. W. L. Jones, T. J. Jones, J. Jongmanns, P. M. Jorge, K. D. Joshi, J. Jovicevic, X. Ju, C. A. Jung, R. M. Jungst, P. Jussel, A. Juste Rozas, M. Kaci, A. Kaczmarska, M. Kado, H. Kagan, M. Kagan, E. Kajomovitz, C. W. Kalderon, S. Kama, A. Kamenshchikov, N. Kanaya, M. Kaneda, S. Kaneti, V. A. Kantserov, J. Kanzaki, B. Kaplan, A. Kapliy, D. Kar, K. Karakostas, N. Karastathis, M. Karnevskiy, S. N. Karpov, Z. M. Karpova, K. Karthik, V. Kartvelishvili, A. N. Karyukhin, L. Kashif, G. Kasieczka, R. D. Kass, A. Kastanas, Y. Kataoka, A. Katre, J. Katzy, V. Kaushik, K. Kawagoe, T. Kawamoto, G. Kawamura, S. Kazama, V. F. Kazanin, M. Y. Kazarinov, R. Keeler, R. Kehoe, M. Keil, J. S. Keller, J. J. Kempster, H. Keoshkerian, O. Kepka, B. P. Kerševan, S. Kersten, K. Kessoku, J. Keung, F. Khalil-zada, H. Khandanyan, A. Khanov, A. Khodinov, A. Khomich, T. J. Khoo, G. Khoriauli, A. Khoroshilov, V. Khovanskiy, E. Khramov, J. Khubua, H. Y. Kim, H. Kim, S. H. Kim, N. Kimura, O. Kind, B. T. King, M. King, R. S. B. King, S. B. King, J. Kirk, A. E. Kiryunin, T. Kishimoto, D. Kisielewska, F. Kiss, T. Kittelmann, K. Kiuchi, E. Kladiva, M. Klein, U. Klein, K. Kleinknecht, P. Klimek, A. Klimentov, R. Klingenberg, J. A. Klinger, T. Klioutchnikova, P. F. Klok, E.-E. Kluge, P. Kluit, S. Kluth, E. Kneringer, E. B. F. G. Knoops, A. Knue, D. Kobayashi, T. Kobayashi, M. Kobel, M. Kocian, P. Kodys, P. Koevesarki, T. Koffas, E. Koffeman, L. A. Kogan, S. Kohlmann, Z. Kohout, T. Kohriki, T. Koi, H. Kolanoski, I. Koletsou, J. Koll, A. A. Komar, Y. Komori, T. Kondo, N. Kondrashova, K. Köneke, A. C. König, S. König, T. Kono, R. Konoplich, N. Konstantinidis, R. Kopeliansky, S. Koperny, L. Köpke, A. K. Kopp, K. Korcyl, K. Kordas, A. Korn, A. A. Korol, I. Korolkov, E. V. Korolkova, V. A. Korotkov, O. Kortner, S. Kortner, V. V. Kostyukhin, V. M. Kotov, A. Kotwal, C. Kourkoumelis, V. Kouskoura, A. Koutsman, R. Kowalewski, T. Z. Kowalski, W. Kozanecki, A. S. Kozhin, V. Kral, V. A. Kramarenko, G. Kramberger, D. Krasnopevtsev, M. W. Krasny, A. Krasznahorkay, J. K. Kraus, A. Kravchenko, S. Kreiss, M. Kretz, J. Kretzschmar, K. Kreutzfeldt, P. Krieger, K. Kroeninger, H. Kroha, J. Kroll, J. Kroseberg, J. Krstic, U. Kruchonak, H. Krüger, T. Kruker, N. Krumnack, Z. V. Krumshteyn, A. Kruse, M. C. Kruse, M. Kruskal, T. Kubota, S. Kuday, S. Kuehn, A. Kugel, A. Kuhl, T. Kuhl, V. Kukhtin, Y. Kulchitsky, S. Kuleshov, M. Kuna, J. Kunkle, A. Kupco, H. Kurashige, Y. A. Kurochkin, R. Kurumida, V. Kus, E. S. Kuwertz, M. Kuze, J. Kvita, A. La Rosa, L. La Rotonda, C. Lacasta, F. Lacava, J. Lacey, H. Lacker, D. Lacour, V. R. Lacuesta, E. Ladygin, R. Lafaye, B. Laforge, T. Lagouri, S. Lai, H. Laier, L. Lambourne, S. Lammers, C. L. Lampen, W. Lampl, E. Lançon, U. Landgraf, M. P. J. Landon, V. S. Lang, A. J. Lankford, F. Lanni, K. Lantzsch, S. Laplace, C. Lapoire, J. F. Laporte, T. Lari, M. Lassnig, P. Laurelli, W. Lavrijsen, A. T. Law, P. Laycock, O. Le Dortz, E. Le Guirriec, E. Le Menedeu, T. LeCompte, F. Ledroit-Guillon, C. A. Lee, H. Lee, J. S. H. Lee, S. C. Lee, L. Lee, G. Lefebvre, M. Lefebvre, F. Legger, C. Leggett, A. Lehan, M. Lehmacher, G. Lehmann Miotto, X. Lei, W. A. Leight, A. Leisos, A. G. Leister, M. A. L. Leite, R. Leitner, D. Lellouch, B. Lemmer, K. J. C. Leney, T. Lenz, G. Lenzen, B. Lenzi, R. Leone, S. Leone, K. Leonhardt, C. Leonidopoulos, S. Leontsinis, C. Leroy, C. G. Lester, C. M. Lester, M. Levchenko, J. Levêque, D. Levin, L. J. Levinson, M. Levy, A. Lewis, G. H. Lewis, A. M. Leyko, M. Leyton, B. Li, B. Li, H. Li, H. L. Li, L. Li, L. Li, S. Li, Y. Li, Z. Liang, H. Liao, B. Liberti, P. Lichard, K. Lie, J. Liebal, W. Liebig, C. Limbach, A. Limosani, S. C. Lin, T. H. Lin, F. Linde, B. E. Lindquist, J. T. Linnemann, E. Lipeles, A. Lipniacka, M. Lisovyi, T. M. Liss, D. Lissauer, A. Lister, A. M. Litke, B. Liu, D. Liu, J. B. Liu, K. Liu, L. Liu, M. Liu, M. Liu, Y. Liu, M. Livan, S. S. A. Livermore, A. Lleres, J. Llorente Merino, S. L. Lloyd, F. Lo Sterzo, E. Lobodzinska, P. Loch, W. S. Lockman, T. Loddenkoetter, F. K. Loebinger, A. E. Loevschall-Jensen, A. Loginov, T. Lohse, K. Lohwasser, M. Lokajicek, V. P. Lombardo, B. A. Long, J. D. Long, R. E. Long, L. Lopes, D. Lopez Mateos, B. Lopez Paredes, I. Lopez Paz, J. Lorenz, N. Lorenzo Martinez, M. Losada, P. Loscutoff, X. Lou, A. Lounis, J. Love, P. A. Love, A. J. Lowe, F. Lu, N. Lu, H. J. Lubatti, C. Luci, A. Lucotte, F. Luehring, W. Lukas, L. Luminari, O. Lundberg, B. Lund-Jensen, M. Lungwitz, D. Lynn, R. Lysak, E. Lytken, H. Ma, L. L. Ma, G. Maccarrone, A. Macchiolo, J. Machado Miguens, D. Macina, D. Madaffari, R. Madar, H. J. Maddocks, W. F. Mader, A. Madsen, M. Maeno, T. Maeno, E. Magradze, K. Mahboubi, J. Mahlstedt, S. Mahmoud, C. Maiani, C. Maidantchik, A. A. Maier, A. Maio, S. Majewski, Y. Makida, N. Makovec, P. Mal, B. Malaescu, Pa. Malecki, V. P. Maleev, F. Malek, U. Mallik, D. Malon, C. Malone, S. Maltezos, V. M. Malyshev, S. Malyukov, J. Mamuzic, B. Mandelli, L. Mandelli, I. Mandić, R. Mandrysch, J. Maneira, A. Manfredini, L. Manhaes de Andrade Filho, J. A. Manjarres Ramos, A. Mann, P. M. Manning, A. Manousakis-Katsikakis, B. Mansoulie, R. Mantifel, L. Mapelli, L. March, J. F. Marchand, G. Marchiori, M. Marcisovsky, C. P. Marino, M. Marjanovic, C. N. Marques, F. Marroquim, S. P. Marsden, Z. Marshall, L. F. Marti, S. Marti-Garcia, B. Martin, B. Martin, T. A. Martin, V. J. Martin, B. Martin dit Latour, H. Martinez, M. Martinez, S. Martin-Haugh, A. C. Martyniuk, M. Marx, F. Marzano, A. Marzin, L. Masetti, T. Mashimo, R. Mashinistov, J. Masik, A. L. Maslennikov, I. Massa, L. Massa, N. Massol, P. Mastrandrea, A. Mastroberardino, T. Masubuchi, P. Mättig, J. Mattmann, J. Maurer, S. J. Maxfield, D. A. Maximov, R. Mazini, L. Mazzaferro, G. Mc Goldrick, S. P. Mc Kee, A. McCarn, R. L. McCarthy, T. G. McCarthy, N. A. McCubbin, K. W. McFarlane, J. A. Mcfayden, G. Mchedlidze, S. J. McMahon, R. A. McPherson, A. Meade, J. Mechnich, M. Medinnis, S. Meehan, S. Mehlhase, A. Mehta, K. Meier, C. Meineck, B. Meirose, C. Melachrinos, B. R. Mellado Garcia, F. Meloni, A. Mengarelli, S. Menke, E. Meoni, K. M. Mercurio, S. Mergelmeyer, N. Meric, P. Mermod, L. Merola, C. Meroni, F. S. Merritt, H. Merritt, A. Messina, J. Metcalfe, A. S. Mete, C. Meyer, C. Meyer, J-P. Meyer, J. Meyer, R. P. Middleton, S. Migas, L. Mijović, G. Mikenberg, M. Mikestikova, M. Mikuž, A. Milic, D. W. Miller, C. Mills, A. Milov, D. A. Milstead, D. Milstein, A. A. Minaenko, I. A. Minashvili, A. I. Mincer, B. Mindur, M. Mineev, Y. Ming, L. M. Mir, G. Mirabelli, T. Mitani, J. Mitrevski, V. A. Mitsou, S. Mitsui, A. Miucci, P. S. Miyagawa, J. U. Mjörnmark, T. Moa, K. Mochizuki, S. Mohapatra, W. Mohr, S. Molander, R. Moles-Valls, K. Mönig, C. Monini, J. Monk, E. Monnier, J. Montejo Berlingen, F. Monticelli, S. Monzani, R. W. Moore, A. Moraes, N. Morange, D. Moreno, M. Moreno Llácer, P. Morettini, M. Morgenstern, M. Morii, S. Moritz, A. K. Morley, G. Mornacchi, J. D. Morris, L. Morvaj, H. G. Moser, M. Mosidze, J. Moss, K. Motohashi, R. Mount, E. Mountricha, S. V. Mouraviev, E. J. W. Moyse, S. Muanza, R. D. Mudd, F. Mueller, J. Mueller, K. Mueller, T. Mueller, T. Mueller, D. Muenstermann, Y. Munwes, J. A. Murillo Quijada, W. J. Murray, H. Musheghyan, E. Musto, A. G. Myagkov, M. Myska, O. Nackenhorst, J. Nadal, K. Nagai, R. Nagai, Y. Nagai, K. Nagano, A. Nagarkar, Y. Nagasaka, M. Nagel, A. M. Nairz, Y. Nakahama, K. Nakamura, T. Nakamura, I. Nakano, H. Namasivayam, G. Nanava, R. Narayan, T. Nattermann, T. Naumann, G. Navarro, R. Nayyar, H. A. Neal, P. Yu. Nechaeva, T. J. Neep, P. D. Nef, A. Negri, G. Negri, M. Negrini, S. Nektarijevic, A. Nelson, T. K. Nelson, S. Nemecek, P. Nemethy, A. A. Nepomuceno, M. Nessi, M. S. Neubauer, M. Neumann, R. M. Neves, P. Nevski, P. R. Newman, D. H. Nguyen, R. B. Nickerson, R. Nicolaidou, B. Nicquevert, J. Nielsen, N. Nikiforou, A. Nikiforov, V. Nikolaenko, I. Nikolic-Audit, K. Nikolics, K. Nikolopoulos, P. Nilsson, Y. Ninomiya, A. Nisati, R. Nisius, T. Nobe, L. Nodulman, M. Nomachi, I. Nomidis, S. Norberg, M. Nordberg, O. Novgorodova, S. Nowak, M. Nozaki, L. Nozka, K. Ntekas, G. Nunes Hanninger, T. Nunnemann, E. Nurse, F. Nuti, B. J. O’Brien, F. O’grady, D. C. O’Neil, V. O’Shea, F. G. Oakham, H. Oberlack, T. Obermann, J. Ocariz, A. Ochi, M. I. Ochoa, S. Oda, S. Odaka, H. Ogren, A. Oh, S. H. Oh, C. C. Ohm, H. Ohman, W. Okamura, H. Okawa, Y. Okumura, T. Okuyama, A. Olariu, A. G. Olchevski, S. A. Olivares Pino, D. Oliveira Damazio, E. Oliver Garcia, A. Olszewski, J. Olszowska, A. Onofre, P. U. E. Onyisi, C. J. Oram, M. J. Oreglia, Y. Oren, D. Orestano, N. Orlando, C. Oropeza Barrera, R. S. Orr, B. Osculati, R. Ospanov, G. Otero y Garzon, H. Otono, M. Ouchrif, E. A. Ouellette, F. Ould-Saada, A. Ouraou, K. P. Oussoren, Q. Ouyang, A. Ovcharova, M. Owen, V. E. Ozcan, N. Ozturk, K. Pachal, A. Pacheco Pages, C. Padilla Aranda, M. Pagáčová, S. Pagan Griso, E. Paganis, C. Pahl, F. Paige, P. Pais, K. Pajchel, G. Palacino, S. Palestini, M. Palka, D. Pallin, A. Palma, J. D. Palmer, Y. B. Pan, E. Panagiotopoulou, J. G. Panduro Vazquez, P. Pani, N. Panikashvili, S. Panitkin, D. Pantea, L. Paolozzi, Th. D. Papadopoulou, K. Papageorgiou, A. Paramonov, D. Paredes Hernandez, M. A. Parker, F. Parodi, J. A. Parsons, U. Parzefall, E. Pasqualucci, S. Passaggio, A. Passeri, F. Pastore, Fr. Pastore, G. Pásztor, S. Pataraia, N. D. Patel, J. R. Pater, S. Patricelli, T. Pauly, J. Pearce, M. Pedersen, S. Pedraza Lopez, R. Pedro, S. V. Peleganchuk, D. Pelikan, H. Peng, B. Penning, J. Penwell, D. V. Perepelitsa, E. Perez Codina, M. T. Pérez García-Estañ, V. Perez Reale, L. Perini, H. Pernegger, R. Perrino, R. Peschke, V. D. Peshekhonov, K. Peters, R. F. Y. Peters, B. A. Petersen, T. C. Petersen, E. Petit, A. Petridis, C. Petridou, E. Petrolo, F. Petrucci, N. E. Pettersson, R. Pezoa, P. W. Phillips, G. Piacquadio, E. Pianori, A. Picazio, E. Piccaro, M. Piccinini, R. Piegaia, D. T. Pignotti, J. E. Pilcher, A. D. Pilkington, J. Pina, M. Pinamonti, A. Pinder, J. L. Pinfold, A. Pingel, B. Pinto, S. Pires, M. Pitt, C. Pizio, L. Plazak, M.-A. Pleier, V. Pleskot, E. Plotnikova, P. Plucinski, S. Poddar, F. Podlyski, R. Poettgen, L. Poggioli, D. Pohl, M. Pohl, G. Polesello, A. Policicchio, R. Polifka, A. Polini, C. S. Pollard, V. Polychronakos, K. Pommès, L. Pontecorvo, B. G. Pope, G. A. Popeneciu, D. S. Popovic, A. Poppleton, X. Portell Bueso, S. Pospisil, K. Potamianos, I. N. Potrap, C. J. Potter, C. T. Potter, G. Poulard, J. Poveda, V. Pozdnyakov, P. Pralavorio, A. Pranko, S. Prasad, R. Pravahan, S. Prell, D. Price, J. Price, L. E. Price, D. Prieur, M. Primavera, M. Proissl, K. Prokofiev, F. Prokoshin, E. Protopapadaki, S. Protopopescu, J. Proudfoot, M. Przybycien, H. Przysiezniak, E. Ptacek, D. Puddu, E. Pueschel, D. Puldon, M. Purohit, P. Puzo, J. Qian, G. Qin, Y. Qin, A. Quadt, D. R. Quarrie, W. B. Quayle, M. Queitsch-Maitland, D. Quilty, A. Qureshi, V. Radeka, V. Radescu, S. K. Radhakrishnan, P. Radloff, P. Rados, F. Ragusa, G. Rahal, S. Rajagopalan, M. Rammensee, A. S. Randle-Conde, C. Rangel-Smith, K. Rao, F. Rauscher, T. C. Rave, T. Ravenscroft, M. Raymond, A. L. Read, N. P. Readioff, D. M. Rebuzzi, A. Redelbach, G. Redlinger, R. Reece, K. Reeves, L. Rehnisch, H. Reisin, M. Relich, C. Rembser, H. Ren, Z. L. Ren, A. Renaud, M. Rescigno, S. Resconi, O. L. Rezanova, P. Reznicek, R. Rezvani, R. Richter, M. Ridel, P. Rieck, J. Rieger, M. Rijssenbeek, A. Rimoldi, L. Rinaldi, E. Ritsch, I. Riu, F. Rizatdinova, E. Rizvi, S. H. Robertson, A. Robichaud-Veronneau, D. Robinson, J. E. M. Robinson, A. Robson, C. Roda, L. Rodrigues, S. Roe, O. Røhne, S. Rolli, A. Romaniouk, M. Romano, E. Romero Adam, N. Rompotis, M. Ronzani, L. Roos, E. Ros, S. Rosati, K. Rosbach, M. Rose, P. Rose, P. L. Rosendahl, O. Rosenthal, V. Rossetti, E. Rossi, L. P. Rossi, R. Rosten, M. Rotaru, I. Roth, J. Rothberg, D. Rousseau, C. R. Royon, A. Rozanov, Y. Rozen, X. Ruan, F. Rubbo, I. Rubinskiy, V. I. Rud, C. Rudolph, M. S. Rudolph, F. Rühr, A. Ruiz-Martinez, Z. Rurikova, N. A. Rusakovich, A. Ruschke, J. P. Rutherfoord, N. Ruthmann, Y. F. Ryabov, M. Rybar, G. Rybkin, N. C. Ryder, A. F. Saavedra, S. Sacerdoti, A. Saddique, I. Sadeh, H.F-W. Sadrozinski, R. Sadykov, F. Safai Tehrani, H. Sakamoto, Y. Sakurai, G. Salamanna, A. Salamon, M. Saleem, D. Salek, P. H. Sales De Bruin, D. Salihagic, A. Salnikov, J. Salt, D. Salvatore, F. Salvatore, A. Salvucci, A. Salzburger, D. Sampsonidis, A. Sanchez, J. Sánchez, V. Sanchez Martinez, H. Sandaker, R. L. Sandbach, H. G. Sander, M. P. Sanders, M. Sandhoff, T. Sandoval, C. Sandoval, R. Sandstroem, D. P. C. Sankey, A. Sansoni, C. Santoni, R. Santonico, H. Santos, I. Santoyo Castillo, K. Sapp, A. Sapronov, J. G. Saraiva, B. Sarrazin, G. Sartisohn, O. Sasaki, Y. Sasaki, G. Sauvage, E. Sauvan, P. Savard, D. O. Savu, C. Sawyer, L. Sawyer, D. H. Saxon, J. Saxon, C. Sbarra, A. Sbrizzi, T. Scanlon, D. A. Scannicchio, M. Scarcella, V. Scarfone, J. Schaarschmidt, P. Schacht, D. Schaefer, R. Schaefer, S. Schaepe, S. Schaetzel, U. Schäfer, A. C. Schaffer, D. Schaile, R. D. Schamberger, V. Scharf, V. A. Schegelsky, D. Scheirich, M. Schernau, M. I. Scherzer, C. Schiavi, J. Schieck, C. Schillo, M. Schioppa, S. Schlenker, E. Schmidt, K. Schmieden, C. Schmitt, S. Schmitt, B. Schneider, Y. J. Schnellbach, U. Schnoor, L. Schoeffel, A. Schoening, B. D. Schoenrock, A. L. S. Schorlemmer, M. Schott, D. Schouten, J. Schovancova, S. Schramm, M. Schreyer, C. Schroeder, N. Schuh, M. J. Schultens, H.-C. Schultz-Coulon, H. Schulz, M. Schumacher, B. A. Schumm, Ph. Schune, C. Schwanenberger, A. Schwartzman, Ph. Schwegler, Ph. Schwemling, R. Schwienhorst, J. Schwindling, T. Schwindt, M. Schwoerer, F. G. Sciacca, E. Scifo, G. Sciolla, W. G. Scott, F. Scuri, F. Scutti, J. Searcy, G. Sedov, E. Sedykh, S. C. Seidel, A. Seiden, F. Seifert, J. M. Seixas, G. Sekhniaidze, S. J. Sekula, K. E. Selbach, D. M. Seliverstov, G. Sellers, N. Semprini-Cesari, C. Serfon, L. Serin, L. Serkin, T. Serre, R. Seuster, H. Severini, T. Sfiligoj, F. Sforza, A. Sfyrla, E. Shabalina, M. Shamim, L. Y. Shan, R. Shang, J. T. Shank, M. Shapiro, P. B. Shatalov, K. Shaw, C. Y. Shehu, P. Sherwood, L. Shi, S. Shimizu, C. O. Shimmin, M. Shimojima, M. Shiyakova, A. Shmeleva, M. J. Shochet, D. Short, S. Shrestha, E. Shulga, M. A. Shupe, S. Shushkevich, P. Sicho, O. Sidiropoulou, D. Sidorov, A. Sidoti, F. Siegert, Dj. Sijacki, J. Silva, Y. Silver, D. Silverstein, S. B. Silverstein, V. Simak, O. Simard, Lj. Simic, S. Simion, E. Simioni, B. Simmons, R. Simoniello, M. Simonyan, P. Sinervo, N. B. Sinev, V. Sipica, G. Siragusa, A. Sircar, A. N. Sisakyan, S. Yu. Sivoklokov, J. Sjölin, T. B. Sjursen, H. P. Skottowe, K. Yu. Skovpen, P. Skubic, M. Slater, T. Slavicek, K. Sliwa, V. Smakhtin, B. H. Smart, L. Smestad, S. Yu. Smirnov, Y. Smirnov, L. N. Smirnova, O. Smirnova, K. M. Smith, M. Smizanska, K. Smolek, A. A. Snesarev, G. Snidero, S. Snyder, R. Sobie, F. Socher, A. Soffer, D. A. Soh, C. A. Solans, M. Solar, J. Solc, E. Yu. Soldatov, U. Soldevila, A. A. Solodkov, A. Soloshenko, O. V. Solovyanov, V. Solovyev, P. Sommer, H. Y. Song, N. Soni, A. Sood, A. Sopczak, B. Sopko, V. Sopko, V. Sorin, M. Sosebee, R. Soualah, P. Soueid, A. M. Soukharev, D. South, S. Spagnolo, F. Spanò, W. R. Spearman, F. Spettel, R. Spighi, G. Spigo, L. A. Spiller, M. Spousta, T. Spreitzer, B. Spurlock, R. D. St. Denis, S. Staerz, J. Stahlman, R. Stamen, S. Stamm, E. Stanecka, R. W. Stanek, C. Stanescu, M. Stanescu-Bellu, M. M. Stanitzki, S. Stapnes, E. A. Starchenko, J. Stark, P. Staroba, P. Starovoitov, R. Staszewski, P. Stavina, P. Steinberg, B. Stelzer, H. J. Stelzer, O. Stelzer-Chilton, H. Stenzel, S. Stern, G. A. Stewart, J. A. Stillings, M. C. Stockton, M. Stoebe, G. Stoicea, P. Stolte, S. Stonjek, A. R. Stradling, A. Straessner, M. E. Stramaglia, J. Strandberg, S. Strandberg, A. Strandlie, E. Strauss, M. Strauss, P. Strizenec, R. Ströhmer, D. M. Strom, R. Stroynowski, S. A. Stucci, B. Stugu, N. A. Styles, D. Su, J. Su, R. Subramaniam, A. Succurro, Y. Sugaya, C. Suhr, M. Suk, V. V. Sulin, S. Sultansoy, T. Sumida, S. Sun, X. Sun, J. E. Sundermann, K. Suruliz, G. Susinno, M. R. Sutton, Y. Suzuki, M. Svatos, S. Swedish, M. Swiatlowski, I. Sykora, T. Sykora, D. Ta, C. Taccini, K. Tackmann, J. Taenzer, A. Taffard, R. Tafirout, N. Taiblum, H. Takai, R. Takashima, H. Takeda, T. Takeshita, Y. Takubo, M. Talby, A. A. Talyshev, J. Y. C. Tam, K. G. Tan, J. Tanaka, R. Tanaka, S. Tanaka, S. Tanaka, A. J. Tanasijczuk, B. B. Tannenwald, N. Tannoury, S. Tapprogge, S. Tarem, F. Tarrade, G. F. Tartarelli, P. Tas, M. Tasevsky, T. Tashiro, E. Tassi, A. Tavares Delgado, Y. Tayalati, F. E. Taylor, G. N. Taylor, W. Taylor, F. A. Teischinger, M. Teixeira Dias Castanheira, P. Teixeira-Dias, K. K. Temming, H. Ten Kate, P. K. Teng, J. J. Teoh, S. Terada, K. Terashi, J. Terron, S. Terzo, M. Testa, R. J. Teuscher, J. Therhaag, T. Theveneaux-Pelzer, J. P. Thomas, J. Thomas-Wilsker, E. N. Thompson, P. D. Thompson, P. D. Thompson, R. J. Thompson, A. S. Thompson, L. A. Thomsen, E. Thomson, M. Thomson, W. M. Thong, R. P. Thun, F. Tian, M. J. Tibbetts, V. O. Tikhomirov, Yu. A. Tikhonov, S. Timoshenko, E. Tiouchichine, P. Tipton, S. Tisserant, T. Todorov, S. Todorova-Nova, B. Toggerson, J. Tojo, S. Tokár, K. Tokushuku, K. Tollefson, L. Tomlinson, M. Tomoto, L. Tompkins, K. Toms, N. D. Topilin, E. Torrence, H. Torres, E. Torró Pastor, J. Toth, F. Touchard, D. R. Tovey, H. L. Tran, T. Trefzger, L. Tremblet, A. Tricoli, I. M. Trigger, S. Trincaz-Duvoid, M. F. Tripiana, W. Trischuk, B. Trocmé, C. Troncon, M. Trottier-McDonald, M. Trovatelli, P. True, M. Trzebinski, A. Trzupek, C. Tsarouchas, J.C-L. Tseng, P. V. Tsiareshka, D. Tsionou, G. Tsipolitis, N. Tsirintanis, S. Tsiskaridze, V. Tsiskaridze, E. G. Tskhadadze, I. I. Tsukerman, V. Tsulaia, S. Tsuno, D. Tsybychev, A. Tudorache, V. Tudorache, A. N. Tuna, S. A. Tupputi, S. Turchikhin, D. Turecek, I. Turk Cakir, R. Turra, P. M. Tuts, A. Tykhonov, M. Tylmad, M. Tyndel, K. Uchida, I. Ueda, R. Ueno, M. Ughetto, M. Ugland, M. Uhlenbrock, F. Ukegawa, G. Unal, A. Undrus, G. Unel, F. C. Ungaro, Y. Unno, C. Unverdorben, D. Urbaniec, P. Urquijo, G. Usai, A. Usanova, L. Vacavant, V. Vacek, B. Vachon, N. Valencic, S. Valentinetti, A. Valero, L. Valery, S. Valkar, E. Valladolid Gallego, S. Vallecorsa, J. A. Valls Ferrer, W. Van Den Wollenberg, P. C. Van Der Deijl, R. van der Geer, H. van der Graaf, R. Van Der Leeuw, D. van der Ster, N. van Eldik, P. van Gemmeren, J. Van Nieuwkoop, I. van Vulpen, M. C. van Woerden, M. Vanadia, W. Vandelli, R. Vanguri, A. Vaniachine, P. Vankov, F. Vannucci, G. Vardanyan, R. Vari, E. W. Varnes, T. Varol, D. Varouchas, A. Vartapetian, K. E. Varvell, F. Vazeille, T. Vazquez Schroeder, J. Veatch, F. Veloso, S. Veneziano, A. Ventura, D. Ventura, M. Venturi, N. Venturi, A. Venturini, V. Vercesi, M. Verducci, W. Verkerke, J. C. Vermeulen, A. Vest, M. C. Vetterli, O. Viazlo, I. Vichou, T. Vickey, O. E. Vickey Boeriu, G. H. A. Viehhauser, S. Viel, R. Vigne, M. Villa, M. Villaplana Perez, E. Vilucchi, M. G. Vincter, V. B. Vinogradov, J. Virzi, I. Vivarelli, F. Vives Vaque, S. Vlachos, D. Vladoiu, M. Vlasak, A. Vogel, M. Vogel, P. Vokac, G. Volpi, M. Volpi, H. von der Schmitt, H. von Radziewski, E. von Toerne, V. Vorobel, K. Vorobev, M. Vos, R. Voss, J. H. Vossebeld, N. Vranjes, M. Vranjes Milosavljevic, V. Vrba, M. Vreeswijk, T. Vu Anh, R. Vuillermet, I. Vukotic, Z. Vykydal, P. Wagner, W. Wagner, H. Wahlberg, S. Wahrmund, J. Wakabayashi, J. Walder, R. Walker, W. Walkowiak, R. Wall, P. Waller, B. Walsh, C. Wang, C. Wang, F. Wang, H. Wang, H. Wang, J. Wang, J. Wang, K. Wang, R. Wang, S. M. Wang, T. Wang, X. Wang, C. Wanotayaroj, A. Warburton, C. P. Ward, D. R. Wardrope, M. Warsinsky, A. Washbrook, C. Wasicki, P. M. Watkins, A. T. Watson, I. J. Watson, M. F. Watson, G. Watts, S. Watts, B. M. Waugh, S. Webb, M. S. Weber, S. W. Weber, J. S. Webster, A. R. Weidberg, P. Weigell, B. Weinert, J. Weingarten, C. Weiser, H. Weits, P. S. Wells, T. Wenaus, D. Wendland, Z. Weng, T. Wengler, S. Wenig, N. Wermes, M. Werner, P. Werner, M. Wessels, J. Wetter, K. Whalen, A. White, M. J. White, R. White, S. White, D. Whiteson, D. Wicke, F. J. Wickens, W. Wiedenmann, M. Wielers, P. Wienemann, C. Wiglesworth, L. A. M. Wiik-Fuchs, P. A. Wijeratne, A. Wildauer, M. A. Wildt, H. G. Wilkens, J. Z. Will, H. H. Williams, S. Williams, C. Willis, S. Willocq, A. Wilson, J. A. Wilson, I. Wingerter-Seez, F. Winklmeier, B. T. Winter, M. Wittgen, T. Wittig, J. Wittkowski, S. J. Wollstadt, M. W. Wolter, H. Wolters, B. K. Wosiek, J. Wotschack, M. J. Woudstra, K. W. Wozniak, M. Wright, M. Wu, S. L. Wu, X. Wu, Y. Wu, E. Wulf, T. R. Wyatt, B. M. Wynne, S. Xella, M. Xiao, D. Xu, L. Xu, B. Yabsley, S. Yacoob, R. Yakabe, M. Yamada, H. Yamaguchi, Y. Yamaguchi, A. Yamamoto, K. Yamamoto, S. Yamamoto, T. Yamamura, T. Yamanaka, K. Yamauchi, Y. Yamazaki, Z. Yan, H. Yang, H. Yang, U. K. Yang, Y. Yang, S. Yanush, L. Yao, W-M. Yao, Y. Yasu, E. Yatsenko, K. H. Yau Wong, J. Ye, S. Ye, I. Yeletskikh, A. L. Yen, E. Yildirim, M. Yilmaz, R. Yoosoofmiya, K. Yorita, R. Yoshida, K. Yoshihara, C. Young, C. J. S. Young, S. Youssef, D. R. Yu, J. Yu, J. M. Yu, J. Yu, L. Yuan, A. Yurkewicz, I. Yusuff, B. Zabinski, R. Zaidan, A. M. Zaitsev, A. Zaman, S. Zambito, L. Zanello, D. Zanzi, C. Zeitnitz, M. Zeman, A. Zemla, K. Zengel, O. Zenin, T. Ženiš, D. Zerwas, G. Zevi della Porta, D. Zhang, F. Zhang, H. Zhang, J. Zhang, L. Zhang, X. Zhang, Z. Zhang, Z. Zhao, A. Zhemchugov, J. Zhong, B. Zhou, L. Zhou, N. Zhou, C. G. Zhu, H. Zhu, J. Zhu, Y. Zhu, X. Zhuang, K. Zhukov, A. Zibell, D. Zieminska, N. I. Zimine, C. Zimmermann, R. Zimmermann, S. Zimmermann, S. Zimmermann, Z. Zinonos, M. Ziolkowski, G. Zobernig, A. Zoccoli, M. zur Nedden, G. Zurzolo, V. Zutshi, L. Zwalinski

**Affiliations:** Department of Physics, University of Adelaide, Adelaide, Australia; Physics Department, SUNY Albany, Albany, NY USA; Department of Physics, University of Alberta, Edmonton, AB Canada; Department of Physics, Ankara University, Ankara, Turkey; Department of Physics, Gazi University, Ankara, Turkey; Division of Physics, TOBB University of Economics and Technology, Ankara, Turkey; Turkish Atomic Energy Authority, Ankara, Turkey; LAPP, CNRS/IN2P3 and Université de Savoie, Annecy-le-Vieux, France; High Energy Physics Division, Argonne National Laboratory, Argonne, IL USA; Department of Physics, University of Arizona, Tucson, AZ USA; Department of Physics, The University of Texas at Arlington, Arlington, TX USA; Physics Department, University of Athens, Athens, Greece; Physics Department, National Technical University of Athens, Zografou, Greece; Institute of Physics, Azerbaijan Academy of Sciences, Baku, Azerbaijan; Institut de Física d’Altes Energies and Departament de Física de la Universitat Autònoma de Barcelona, Barcelona, Spain; Institute of Physics, University of Belgrade, Belgrade, Serbia; Vinca Institute of Nuclear Sciences, University of Belgrade, Belgrade, Serbia; Department for Physics and Technology, University of Bergen, Bergen, Norway; Physics Division, Lawrence Berkeley National Laboratory and University of California, Berkeley, CA USA; Department of Physics, Humboldt University, Berlin, Germany; Albert Einstein Center for Fundamental Physics and Laboratory for High Energy Physics, University of Bern, Bern, Switzerland; School of Physics and Astronomy, University of Birmingham, Birmingham, UK; Department of Physics, Bogazici University, Istanbul, Turkey; Department of Physics, Dogus University, Istanbul, Turkey; Department of Physics Engineering, Gaziantep University, Gaziantep, Turkey; INFN Sezione di Bologna, Bologna, Italy; Dipartimento di Fisica e Astronomia, Università di Bologna, Bologna, Italy; Physikalisches Institut, University of Bonn, Bonn, Germany; Department of Physics, Boston University, Boston, MA USA; Department of Physics, Brandeis University, Waltham, MA USA; Universidade Federal do Rio De Janeiro COPPE/EE/IF, Rio de Janeiro, Brazil; Federal University of Juiz de Fora (UFJF), Juiz de Fora, Brazil; Federal University of Sao Joao del Rei (UFSJ), Sao Joao del Rei, Brazil; Instituto de Fisica, Universidade de Sao Paulo, Sao Paulo, Brazil; Physics Department, Brookhaven National Laboratory, Upton, NY USA; National Institute of Physics and Nuclear Engineering, Bucharest, Romania; Physics Department, National Institute for Research and Development of Isotopic and Molecular Technologies, Cluj Napoca, Romania; University Politehnica Bucharest, Bucharest, Romania; West University in Timisoara, Timisoara, Romania; Departamento de Física, Universidad de Buenos Aires, Buenos Aires, Argentina; Cavendish Laboratory, University of Cambridge, Cambridge, UK; Department of Physics, Carleton University, Ottawa, ON Canada; CERN, Geneva, Switzerland; Enrico Fermi Institute, University of Chicago, Chicago, IL USA; Departamento de Física, Pontificia Universidad Católica de Chile, Santiago, Chile; Departamento de Física, Universidad Técnica Federico Santa María, Valparaíso, Chile; Institute of High Energy Physics, Chinese Academy of Sciences, Beijing, China; Department of Modern Physics, University of Science and Technology of China, Anhui, China; Department of Physics, Nanjing University, Jiangsu, China; School of Physics, Shandong University, Shandong, China; Physics Department, Shanghai Jiao Tong University, Shanghai, China; Laboratoire de Physique Corpusculaire, Clermont Université and Université Blaise Pascal and CNRS/IN2P3, Clermont-Ferrand, France; Nevis Laboratory, Columbia University, Irvington, NY USA; Niels Bohr Institute, University of Copenhagen, Kobenhavn, Denmark; INFN Gruppo Collegato di Cosenza, Laboratori Nazionali di Frascati, Italy; Dipartimento di Fisica, Università della Calabria, Rende, Italy; Faculty of Physics and Applied Computer Science, AGH University of Science and Technology, Kraków, Poland; Marian Smoluchowski Institute of Physics, Jagiellonian University, Kraków, Poland; The Henryk Niewodniczanski Institute of Nuclear Physics, Polish Academy of Sciences, Kraków, Poland; Physics Department, Southern Methodist University, Dallas, TX USA; Physics Department, University of Texas at Dallas, Richardson, TX USA; DESY, Hamburg and Zeuthen, Germany; Institut für Experimentelle Physik IV, Technische Universität Dortmund, Dortmund, Germany; Institut für Kern- und Teilchenphysik, Technische Universität Dresden, Dresden, Germany; Department of Physics, Duke University, Durham, NC USA; SUPA-School of Physics and Astronomy, University of Edinburgh, Edinburgh, UK; INFN Laboratori Nazionali di Frascati, Frascati, Italy; Fakultät für Mathematik und Physik, Albert-Ludwigs-Universität, Freiburg, Germany; Section de Physique, Université de Genève, Geneva, Switzerland; INFN Sezione di Genova, Genoa, Italy; Dipartimento di Fisica, Università di Genova, Genoa, Italy; E. Andronikashvili Institute of Physics, Iv. Javakhishvili Tbilisi State University, Tbilisi, Georgia; High Energy Physics Institute, Tbilisi State University, Tbilisi, Georgia; II Physikalisches Institut, Justus-Liebig-Universität Giessen, Giessen, Germany; SUPA-School of Physics and Astronomy, University of Glasgow, Glasgow, UK; II Physikalisches Institut, Georg-August-Universität, Göttingen, Germany; Laboratoire de Physique Subatomique et de Cosmologie, Université Grenoble-Alpes, CNRS/IN2P3, Grenoble, France; Department of Physics, Hampton University, Hampton, VA USA; Laboratory for Particle Physics and Cosmology, Harvard University, Cambridge, MA USA; Kirchhoff-Institut für Physik, Ruprecht-Karls-Universität Heidelberg, Heidelberg, Germany; Physikalisches Institut, Ruprecht-Karls-Universität Heidelberg, Heidelberg, Germany; ZITI Institut für technische Informatik, Ruprecht-Karls-Universität Heidelberg, Mannheim, Germany; Faculty of Applied Information Science, Hiroshima Institute of Technology, Hiroshima, Japan; Department of Physics, Indiana University, Bloomington, IN USA; Institut für Astro- und Teilchenphysik, Leopold-Franzens-Universität, Innsbruck, Austria; University of Iowa, Iowa City, IA USA; Department of Physics and Astronomy, Iowa State University, Ames, IA USA; Joint Institute for Nuclear Research, JINR Dubna, Dubna, Russia; KEK, High Energy Accelerator Research Organization, Tsukuba, Japan; Graduate School of Science, Kobe University, Kobe, Japan; Faculty of Science, Kyoto University, Kyoto, Japan; Kyoto University of Education, Kyoto, Japan; Department of Physics, Kyushu University, Fukuoka, Japan; Instituto de Física La Plata, Universidad Nacional de La Plata and CONICET, La Plata, Argentina; Physics Department, Lancaster University, Lancaster, UK; INFN Sezione di Lecce, Lecce, Italy; Dipartimento di Matematica e Fisica, Università del Salento, Lecce, Italy; Oliver Lodge Laboratory, University of Liverpool, Liverpool, UK; Department of Physics, Jožef Stefan Institute and University of Ljubljana, Ljubljana, Slovenia; School of Physics and Astronomy, Queen Mary University of London, London, UK; Department of Physics, Royal Holloway University of London, Surrey, UK; Department of Physics and Astronomy, University College London, London, UK; Louisiana Tech University, Ruston, LA USA; Laboratoire de Physique Nucléaire et de Hautes Energies, UPMC and Université Paris-Diderot and CNRS/IN2P3, Paris, France; Fysiska institutionen, Lunds universitet, Lund, Sweden; Departamento de Fisica Teorica C-15, Universidad Autonoma de Madrid, Madrid, Spain; Institut für Physik, Universität Mainz, Mainz, Germany; School of Physics and Astronomy, University of Manchester, Manchester, UK; CPPM, Aix-Marseille Université and CNRS/IN2P3, Marseille, France; Department of Physics, University of Massachusetts, Amherst, MA USA; Department of Physics, McGill University, Montreal, QC Canada; School of Physics, University of Melbourne, Melbourne, VIC Australia; Department of Physics, The University of Michigan, Ann Arbor, MI USA; Department of Physics and Astronomy, Michigan State University, East Lansing, MI USA; INFN Sezione di Milano, Milan, Italy; Dipartimento di Fisica, Università di Milano, Milan, Italy; B.I. Stepanov Institute of Physics, National Academy of Sciences of Belarus, Minsk, Republic of Belarus; National Scientific and Educational Centre for Particle and High Energy Physics, Minsk, Republic of Belarus; Department of Physics, Massachusetts Institute of Technology, Cambridge, MA USA; Group of Particle Physics, University of Montreal, Montreal, QC Canada; P.N. Lebedev Institute of Physics, Academy of Sciences, Moscow, Russia; Institute for Theoretical and Experimental Physics (ITEP), Moscow, Russia; Moscow Engineering and Physics Institute (MEPhI), Moscow, Russia; D.V. Skobeltsyn Institute of Nuclear Physics, M.V. Lomonosov Moscow State University, Moscow, Russia; Fakultät für Physik, Ludwig-Maximilians-Universität München, Munich, Germany; Max-Planck-Institut für Physik (Werner-Heisenberg-Institut), Munich, Germany; Nagasaki Institute of Applied Science, Nagasaki, Japan; Graduate School of Science and Kobayashi-Maskawa Institute, Nagoya University, Nagoya, Japan; INFN Sezione di Napoli, Naples, Italy; Dipartimento di Fisica, Università di Napoli, Naples, Italy; Department of Physics and Astronomy, University of New Mexico, Albuquerque, NM USA; Institute for Mathematics, Astrophysics and Particle Physics, Radboud University Nijmegen/Nikhef, Nijmegen, The Netherlands; Nikhef National Institute for Subatomic Physics and University of Amsterdam, Amsterdam, The Netherlands; Department of Physics, Northern Illinois University, DeKalb, IL USA; Budker Institute of Nuclear Physics, SB RAS, Novosibirsk, Russia; Department of Physics, New York University, New York, NY USA; Ohio State University, Columbus, OH USA; Faculty of Science, Okayama University, Okayama, Japan; Homer L. Dodge Department of Physics and Astronomy, University of Oklahoma, Norman, OK USA; Department of Physics, Oklahoma State University, Stillwater, OK USA; Palacký University, RCPTM, Olomouc, Czech Republic; Center for High Energy Physics, University of Oregon, Eugene, OR USA; LAL, Université Paris-Sud and CNRS/IN2P3, Orsay, France; Graduate School of Science, Osaka University, Osaka, Japan; Department of Physics, University of Oslo, Oslo, Norway; Department of Physics, Oxford University, Oxford, UK; INFN Sezione di Pavia, Pavia, Italy; Dipartimento di Fisica, Università di Pavia, Pavia, Italy; Department of Physics, University of Pennsylvania, Philadelphia, PA USA; Petersburg Nuclear Physics Institute, Gatchina, Russia; INFN Sezione di Pisa, Pisa, Italy; Dipartimento di Fisica E. Fermi, Università di Pisa, Pisa, Italy; Department of Physics and Astronomy, University of Pittsburgh, Pittsburgh, PA USA; Laboratorio de Instrumentacao e Fisica Experimental de Particulas-LIP, Lisbon, Portugal; Faculdade de Ciências, Universidade de Lisboa, Lisbon, Portugal; Department of Physics, University of Coimbra, Coimbra, Portugal; Centro de Física Nuclear da Universidade de Lisboa, Lisbon, Portugal; Departamento de Fisica, Universidade do Minho, Braga, Portugal; Departamento de Fisica Teorica y del Cosmos and CAFPE, Universidad de Granada, Granada, Spain; Dep Fisica and CEFITEC of Faculdade de Ciencias e Tecnologia, Universidade Nova de Lisboa, Caparica, Portugal; Institute of Physics, Academy of Sciences of the Czech Republic, Praha, Czech Republic; Czech Technical University in Prague, Praha, Czech Republic; Faculty of Mathematics and Physics, Charles University in Praha, Prague, Czech Republic; State Research Center Institute for High Energy Physics, Protvino, Russia; Particle Physics Department, Rutherford Appleton Laboratory, Didcot, UK; Physics Department, University of Regina, Regina, SK Canada; Ritsumeikan University, Kusatsu, Shiga Japan; INFN Sezione di Roma, Roma, Italy; Dipartimento di Fisica, Sapienza Università di Roma, Rome, Italy; INFN Sezione di Roma Tor Vergata, Roma, Italy; Dipartimento di Fisica, Università di Roma Tor Vergata, Rome, Italy; INFN Sezione di Roma Tre, Roma, Italy; Dipartimento di Matematica e Fisica, Università Roma Tre, Rome, Italy; Faculté des Sciences Ain Chock, Réseau Universitaire de Physique des Hautes Energies-Université Hassan II, Casablanca, Morocco; Centre National de l’Energie des Sciences Techniques Nucleaires, Rabat, Morocco; Faculté des Sciences Semlalia, Université Cadi Ayyad, LPHEA-Marrakech, Marrakech, Morocco; Faculté des Sciences, Université Mohamed Premier and LPTPM, Oujda, Morocco; Faculté des Sciences, Université Mohammed V-Agdal, Rabat, Morocco; DSM/IRFU (Institut de Recherches sur les Lois Fondamentales de l’Univers), CEA Saclay (Commissariat à l’Energie Atomique et aux Energies Alternatives), Gif-sur-Yvette, France; Santa Cruz Institute for Particle Physics, University of California Santa Cruz, Santa Cruz, CA USA; Department of Physics, University of Washington, Seattle, WA USA; Department of Physics and Astronomy, University of Sheffield, Sheffield, UK; Department of Physics, Shinshu University, Nagano, Japan; Fachbereich Physik, Universität Siegen, Siegen, Germany; Department of Physics, Simon Fraser University, Burnaby, BC Canada; SLAC National Accelerator Laboratory, Stanford, CA USA; Faculty of Mathematics, Physics and Informatics, Comenius University, Bratislava, Slovak Republic; Department of Subnuclear Physics, Institute of Experimental Physics of the Slovak Academy of Sciences, Kosice, Slovak Republic; Department of Physics, University of Cape Town, Cape Town, South Africa; Department of Physics, University of Johannesburg, Johannesburg, South Africa; School of Physics, University of the Witwatersrand, Johannesburg, South Africa; Department of Physics, Stockholm University, Stockholm, Sweden; The Oskar Klein Centre, Stockholm, Sweden; Physics Department, Royal Institute of Technology, Stockholm, Sweden; Departments of Physics and Astronomy and Chemistry, Stony Brook University, Stony Brook, NY USA; Department of Physics and Astronomy, University of Sussex, Brighton, UK; School of Physics, University of Sydney, Sydney, Australia; Institute of Physics, Academia Sinica, Taipei, Taiwan; Department of Physics, Technion: Israel Institute of Technology, Haifa, Israel; Raymond and Beverly Sackler School of Physics and Astronomy, Tel Aviv University, Tel Aviv, Israel; Department of Physics, Aristotle University of Thessaloniki, Thessaloniki, Greece; International Center for Elementary Particle Physics and Department of Physics, The University of Tokyo, Tokyo, Japan; Graduate School of Science and Technology, Tokyo Metropolitan University, Tokyo, Japan; Department of Physics, Tokyo Institute of Technology, Tokyo, Japan; Department of Physics, University of Toronto, Toronto, ON Canada; TRIUMF, Vancouver, BC, Canada; Department of Physics and Astronomy, York University, Toronto, ON Canada; Faculty of Pure and Applied Sciences, University of Tsukuba, Tsukuba, Japan; Department of Physics and Astronomy, Tufts University, Medford, MA USA; Centro de Investigaciones, Universidad Antonio Narino, Bogota, Colombia; Department of Physics and Astronomy, University of California Irvine, Irvine, CA USA; INFN Gruppo Collegato di Udine, Sezione di Trieste, Udine, Italy; ICTP, Trieste, Italy; Dipartimento di Chimica, Fisica e Ambiente, Università di Udine, Udine, Italy; Department of Physics, University of Illinois, Urbana, IL USA; Department of Physics and Astronomy, University of Uppsala, Uppsala, Sweden; Instituto de Física Corpuscular (IFIC) and Departamento de Física Atómica, Molecular y Nuclear and Departamento de Ingeniería Electrónica and Instituto de Microelectrónica de Barcelona (IMB-CNM), University of Valencia and CSIC, Valencia, Spain; Department of Physics, University of British Columbia, Vancouver, BC Canada; Department of Physics and Astronomy, University of Victoria, Victoria, BC Canada; Department of Physics, University of Warwick, Coventry, UK; Waseda University, Tokyo, Japan; Department of Particle Physics, The Weizmann Institute of Science, Rehovot, Israel; Department of Physics, University of Wisconsin, Madison, WI USA; Fakultät für Physik und Astronomie, Julius-Maximilians-Universität, Würzburg, Germany; Fachbereich C Physik, Bergische Universität Wuppertal, Wuppertal, Germany; Department of Physics, Yale University, New Haven, CT USA; Yerevan Physics Institute, Yerevan, Armenia; Centre de Calcul de l’Institut National de Physique Nucléaire et de Physique des Particules (IN2P3), Villeurbanne, France; CERN, 1211 Geneva 23, Switzerland

**Keywords:** ATLAS, LHC, Proton–proton collisions, Top quark, Top-quark mass, Fully hadronic

## Abstract

The mass of the top quark is measured in a data set corresponding to 4.6 $\text{ fb }^{-1}$ of proton–proton collisions with centre-of-mass energy $\sqrt{s}=7$ TeV collected by the ATLAS detector at the LHC. Events consistent with hadronic decays of top–antitop quark pairs with at least six jets in the final state are selected. The substantial background from multijet production is modelled with data-driven methods that utilise the number of identified $b$-quark jets and the transverse momentum of the sixth leading jet, which have minimal correlation. The top-quark mass is obtained from template fits to the ratio of three-jet to dijet mass. The three-jet mass is calculated from the three jets produced in a top-quark decay. Using these three jets the dijet mass is obtained from the two jets produced in the $W$ boson decay. The top-quark mass obtained from this fit is thus less sensitive to the uncertainty in the energy measurement of the jets. A binned likelihood fit yields a top-quark mass of

$$\begin{aligned} m_{t} = 175.1 \pm 1.4\;\text{(stat.) }\pm 1.2\;\text{(syst.) }\mathrm{\,GeV}. \end{aligned}$$

## Introduction

The top quark is the heaviest known fundamental particle and is unique in many respects. In the Standard Model, its large mass derives from a Yukawa coupling to the Higgs boson [1, 2] close to unity. Thus it plays a critical role in the quantum corrections to the electroweak Higgs potential and possible vacuum instability at high energies (see Ref. [3] for a review). Because of its large mass, the top quark has a lifetime shorter than the typical time scale of hadronisation of coloured quarks to hadrons. Hence, the properties of the top quark can be investigated unaffected from non-perturbative effects occuring in hadronic bound states. However, the hadronisation of the quarks and gluons constituting the jets from the decay products of the top quark introduces an unavoidable sensitivity of the measured top-quark mass on non-perturbative effects. The top-quark mass $m_{t}$, is also an essential parameter in high-precision fits to electroweak observables [4].

The top-quark mass can be determined from decay channels involving hadronic and leptonic decays of the intermediate $W$ boson. For the recent world-average top-quark mass value [5], the highest precision [6–15] comes from measurements using the lepton plus jets final state in the decay of top–antitop pairs ($t\bar{t}$). This channel has a substantial branching fraction and allows a relatively unambiguous assignment of jets to partons from the $t\bar{t}$ decay. Such events are selected using the lepton and neutrino from the decay of a $W$ boson from one member of the top–antitop pair.

Events in which the top–antitop quark pair decays into a fully hadronic final state constitute both the largest branching fraction and a complementary final state for the determination of the top-quark mass. The fully hadronic decay mode has been used in Refs. [10, 11] to measure the top-quark mass from $t\bar{t}$ pairs. This decay mode is used in this analysis to measure the top-quark mass from $t\bar{t}$ pairs produced in proton–proton collisions provided by the LHC, and observed by the ATLAS detector. The major background to this final state, with orders of magnitude larger cross section, is multijet production from proton–proton collisions other than $t\bar{t}$ pairs. Particular experimental attention is required to precisely estimate and control this large background. This analysis employs a data-driven method to form a multijet background prediction. Selected data events are divided into several disjoint regions using two uncorrelated observables, such that $t\bar{t}$ events accumulate only in one of these regions. The background is derived from the other regions, determining both the shape and normalisation of the background distribution in the signal region.

As the top-quark mass is calculated from the measured energy and momentum of reconstructed jets, an accurate understanding of energy and momentum measurements is essential. The dependence of the measured top-quark mass on the jet energy measurement uncertainty is reduced by exploiting the fact that two of the three jets originate from the $W$ boson produced in the top-quark decay and that the $W$-boson mass is known very precisely. The analysis presented in this paper uses the observable $R_{3/2}= m_{jjj}/m_{jj}$ to achieve a cancellation of systematic effects common to the masses of the reconstructed top quark ($m_{jjj}$) and associated $W$ boson ($m_{jj}$).

## The ATLAS detector

The ATLAS detector [16] at the LHC covers nearly the entire solid angle around the collision point. The inner detector (ID), which is located closest to the interaction point, provides charged-particle tracking in the range of $|\eta | < 2.5$ where $\eta$ is the pseudorapidity.^1^ The ID comprises a high-granularity silicon pixel detector, a silicon microstrip tracker and a transition radiation tracker, and is surrounded by a thin superconducting solenoid providing a magnetic field of $2$ T. The electromagnetic and hadronic calorimeters are located outside the solenoid and cover the pseudorapidity range $|\eta | < 4.9$. Within the region $|\eta |<3.2$, electromagnetic calorimetry is provided by barrel and endcap lead/liquid-argon (LAr) sampling calorimeters. Hadronic energy measurements are provided by a steel/scintillator tile calorimeter in the central region and copper/LAr calorimeters in the endcaps. The forward regions are instrumented with copper/LAr and tungsten/LAr calorimeters, optimised for electromagnetic and hadronic energy measurements, respectively. The calorimeter system is surrounded by a muon spectrometer, comprising separate trigger and high-precision tracking chambers. They measure the deflection of muons in a magnetic field with a field integral up to $8$ Tm, generated by one barrel and two endcap superconducting air-core toroids.

A three-level trigger system is used. The first-level trigger is implemented in hardware and uses a subset of detector information to reduce the event rate to a design value of at most $75$ kHz. This is followed by two software-based trigger levels, which together reduce the event rate to a few hundred Hz.

The energy scale and resolution of the electromagnetic and hadronic calorimeter systems [17] as well as the performance of the tracking detector for tagging jets from bottom quarks through the displaced decay vertices of $b$-flavoured hadrons [18–20] are of major importance for the precision of this measurement. Jet energies measured by the electromagnetic and hadronic calorimeters are adjusted using correction factors, obtained from an in situ calibration [17], which depend on pseudorapidity ($\eta$) and transverse momentum ($p_{\mathrm {T}}$).

## Data, simulation, event selection and reconstruction

### Data and simulation

This measurement uses data recorded by the ATLAS detector during 2011 from $7\mathrm{\,TeV}$ proton–proton collisions corresponding to an integrated luminosity of $4.6\,\text{ fb }^{-1}$ [21]. Events were generated using Monte Carlo (MC) programs in order to investigate systematic uncertainties, to correct for systematic effects, and to generate template distributions used for fitting the top-quark mass. A fast simulation of the ATLAS detector response, which is based on full simulation of the tracking detectors and on parameterisations for the electromagnetic and hadronic calorimeter showers [22], was applied to the generated events. For systematic studies a smaller sample of events was processed by a full Geant4 [23] simulation of the ATLAS detector [24]. The agreement between parameterised and full simulation was verified in detail, as described in Ref. [22]. The remaining differences are small and accounted for by a systematic uncertainty. All simulated events were subject to the same selection criteria and reconstructed using the same algorithms applied to data. To generate $t\bar{t}$ events, the MC program Powheg-box [25, 26] was employed, which incorporates a theoretical calculation in next-to-leading-order (NLO) accuracy in the strong coupling $\alpha _S$, with NLO parton distribution functions (PDFs) CT10 [27]. The generated partons are showered and hadronised by Pythia [28]. Adjustable parameters of Pythia are fixed to the values obtained in the Perugia 2011C (P2011C) tune [29]. Signal events were generated assuming seven different top-quark mass values from $165.0$ to $180.0\mathrm{\,GeV}$ in steps of $2.5\mathrm{\,GeV}$, with the largest sample at $172.5\mathrm{\,GeV}$. In addition to the hard collisions leading to the $t\bar{t}$ signal, soft scattering processes between the remnants of the protons can take place. Such processes underlying the signal events are also modelled by Pythia using the tuned parameters from Perugia 2011C. Multiple soft proton–proton collisions can take place between different protons in the same bunch crossing (in-time pile-up) or arise from collisions in preceding or subsequent bunch crossings (out-of-time pile-up) due to the time sensitivity of the detector being longer than the time between bunch crossings. Such multiple inelastic interactions were also generated by Pythia, and are reweighted in the simulation to match the distribution of the number of interactions per bunch crossing measured in the data. This number of interactions ranges from 3 to 17, with an average of $8.7$.

For studies of systematic uncertainties an additional, large sample of signal events was generated at $172.5\mathrm{\,GeV}$, using Powheg-box and Pythia with the Perugia 2012 tune.

### Event selection

**Table 1:** Summary of event selection requirements for signal events

Jet-based trigger
$\ge$ 6 jets with $p_{\mathrm {T}}>30\mathrm{\,GeV}$ and $|\eta |<2.5$
$\ge$ 5 jets with $p_{\mathrm {T}}>55\mathrm{\,GeV}$ and $|\eta |<2.5$
$\Delta R > 0.6$ between pairs of jets with $p_{\mathrm {T}}>30\mathrm{\,GeV}$
Jet vertex fraction JVF $>0.75$
Reject events w. isolated electrons with $E_{\mathrm {T}}>25\mathrm{\,GeV}$
Reject events w. isolated muons with $p_{\mathrm {T}}>20\mathrm{\,GeV}$
Exactly 2 $b$-tagged jets among the four leading jets
Missing transverse momentum significance
$$E_{\mathrm {T}}^{\mathrm {miss}}[\mathrm{GeV}]/\sqrt{H_{\mathrm {T}}[\mathrm{GeV}]}<3$$
Centrality $$\mathcal{C}>0.6$$

A jet-based trigger is used in which the jets are reconstructed in the online trigger system [30]. This jet reconstruction executes the anti-$k_t$ jet algorithm [31] with a radius parameter of $0.4$ using clusters of energy deposition in adjacent calorimeter cells (topological clusters) [32, 33]. At least five jets with a nominal $p_{\mathrm {T}}$ threshold of $30\mathrm{\,GeV}$ are required to trigger and record an event.

Events are selected according to the requirements listed in Table 1 and detailed in the following. Only events with a well-reconstructed primary vertex formed by at least five tracks with $p_{\mathrm {T}}>400\mathrm{\,MeV}$ per track are considered for the analysis, where the primary vertex is the reconstructed vertex with the highest summed $p_{\mathrm {T}}^2$ of associated tracks. Similar to the online trigger system, jets are reconstructed offline by the anti-$k_t$ jet algorithm with a radius parameter of $0.4$ using topological clusters. The jet energies are calibrated following Refs. [34–36]. For the parameterised simulation a dedicated jet energy calibration is used which is obtained in the same manner as for the full simulation. To ensure that events selected by the trigger are on the plateau of the efficiency curve, only events which have at least five jets, each with $p_{\mathrm {T}}>55\mathrm{\,GeV}$, and $\Delta R > 0.6$^2^ between every pair of jets with $p_{\mathrm {T}}>30\mathrm{\,GeV}$ are considered. The measured trigger efficiency of $90\,\%$ agrees with the expectation from simulation to within 5 %. This remaining difference is considered as a source of systematic uncertainty in Sect. 6.

A signal event is required to have at least six jets. Only jets in the central part of the calorimeter ($|\eta |<2.5$) and with $p_{\mathrm {T}}> 30\mathrm{\,GeV}$ are considered for the $t\bar{t}$ mass analysis, but for the background determination the sixth leading jet has a looser requirement of $p_{\mathrm {T}}> 25\mathrm{\,GeV}$. For a jet to be considered, at least 75 % of its summed track $p_{\mathrm {T}}$ must be due to tracks coming from the primary vertex (jet vertex fraction JVF $\,>0.75$). Jets in an event are rejected if an identified electron is closer than $\Delta R=0.2$.

Events with identified isolated electrons with $E_{\mathrm {T}}>25\mathrm{\,GeV}$ or muons with $p_{\mathrm {T}}>20\mathrm{\,GeV}$ are rejected. Details of the lepton identification are given in Refs. [37, 38]. Events are kept for further analysis when at most two of the four leading transverse momentum jets are identified as $b$-tagged jets by a neural network trained on decay vertex properties. The neural network provides an identification efficiency of 70 % for jets from $b$-quarks, a rejection factor of about 130 for jets arising from light partons, and a factor of about 5 for jets arising from $c$-quarks [39]. In the signal region, exactly two of the four leading transverse momentum jets are required to be $b$-tagged by the neural network. Events with mismeasured jet energies or with potential leptonic decays that include neutrinos are removed by requiring a missing transverse momentum significance $E_{\mathrm {T}}^{\mathrm {miss}}[\mathrm{GeV}]/\sqrt{H_{\mathrm {T}}[\mathrm{GeV}]}$ of less than 3. Here $H_{\mathrm {T}}$ is the scalar sum of the transverse momenta of all selected jets in the event. The $E_{\mathrm {T}}^{\mathrm {miss}}$ is obtained as in Ref. [15] as the magnitude of the negative vectorial sum of calorimeter energy deposits projected onto the transverse plane, plus the transverse momenta of identified muons measured by the tracking detector and muon spectrometer. Measured energy deposits in the calorimeters are corrected according to the identified object (high-$p_{\mathrm {T}}$ jet, photon, electron, muon); otherwise energy deposits are calibrated with the local hadronic calibration scheme detailed in Ref. [40]. The contribution from multijet background events is reduced by using the centrality $\mathcal{C}$ of the signal events, which is different from the value in multijet events due to the large top-quark mass. Events are required to have $\mathcal{C}> 0.6$, with

1 $$\begin{aligned} \mathcal{C} = \frac{\sum _j^{\mathrm {jets}}{E_{{\mathrm {T}},j}}}{\sqrt{\left( \sum _j^{\mathrm {jets}}p_j\right) ^2}}\ \ , \end{aligned}$$

where $E_{{\mathrm {T}},j}$ is the scalar transverse energy and $p_j=(E_j,\mathbf {p}_j)$ the four-momentum of the $j^{\mathrm {th}}$ selected jet, and the sum is over all selected jets.

### Reconstruction

**Fig. 1:**
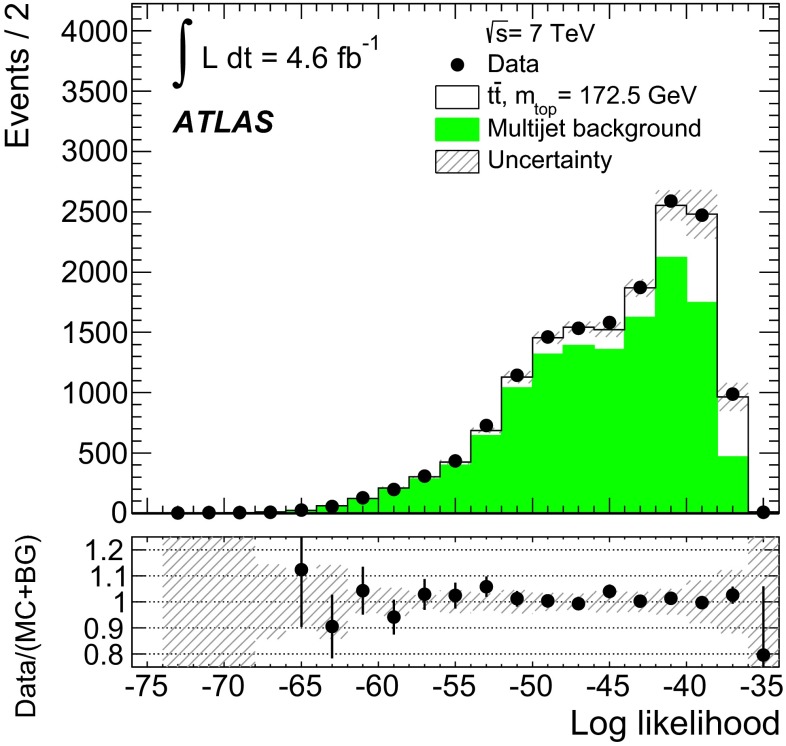
Comparison of the distribution of the unnormalised logarithmic likelihood for the reconstruction of fully hadronic $t\bar{t}$ events in the data with expectations for a top-quark mass value of $172.5\mathrm{\,GeV}$. The graph in the *lower inset* shows the ratio of data to the sum of $t\bar{t}$ MC signal and the modelled multijet background (see Sect. 4). The *error bars* indicate the statistical uncertainty of the data. The *shaded bands* show the statistical and systematic (see Sect. 6) uncertainty on the expected signal and background distributions

In each selected event, a fully hadronic $t\bar{t}$ final state is reconstructed using the six or more jets. In order to achieve this, the jets in data are assigned to the decay partons expected from the decay of the top quark and the related intermediate $W$ boson, assuming a leading-order decay. Exploiting the knowledge of the precisely known mass of the $W$ boson and the Breit–Wigner lineshapes of the top quark and the $W$ boson decay, a kinematic fit [41] based on a likelihood function similar to the one described in Ref. [15] assists in establishing the assignment of reconstructed jets to partons. The fit is performed maximising the logarithmic likelihood, defined as the product of Breit–Wigner distributions for the two top-quark and $W$ boson masses, and MC derived transfer functions for each of the six jets. The Breit–Wigner lineshape functions use the world-average values of the $W$ boson mass ($80.4\mathrm{\,GeV}$) and decay width ($2.1\mathrm{\,GeV}$) from Ref. [42]. The masses of the top quark and antiquark are assumed to be equal for the Breit–Wigner lineshape and free to float in the fit. The top decay width is kept fixed at $1.3\mathrm{\,GeV}$, corresponding to a top-quark mass of $172.5\mathrm{\,GeV}$. The energies of the partons are transferred to the measured jet energies by transfer functions derived from simulation and parameterised by superpositions of two Gaussian functions. It is required, furthermore, that the fit assigns the $b$ and $\overline{b}$ quarks from the $t\bar{t}$ decay to any two of the four leading jets. Maximising the logarithmic likelihood establishes the best assignment of reconstructed jets to partons from the $t\bar{t}$ decay. Figure 1 shows the distribution of the unnormalised logarithmic likelihood value obtained per event and compared with the Monte Carlo prediction of the $t\bar{t}$ signal added to the modelled multijet background (see Sect. 4). The prediction is in good agreement with the shape of the distribution. Requiring the logarithmic likelihood value to be greater than $-45$ removes events which yield a low probability under a $t\bar{t}$ decay hypothesis. The cut rejects about 47 % of the multijet background events, while 79 % of the fully hadronically decaying $t\bar{t}$ events pass the cut.

After applying the above selection requirements and performing the $t\bar{t}$ reconstruction $15 551$ events remain in the signal region for the measurement of the top-quark mass (see Table 2). The expected fraction of $t\bar{t}$ events in this region without any restriction on $R_{3/2}$ is about $17\,\%$, corresponding to a selection efficiency of $\approx$0.5 %.

## Modelling of multijet background

The multijet background contribution is large and cannot be removed completely from any distribution used to measure the top-quark mass in the fully hadronic final state. Currently only leading-order theory calculations for final states with up to six parton are available in MC generator programs. Therefore, the multijet background is determined from the data.

For this approach, selected events are divided into six regions ($A$–$F$) by using two observables with minimal correlation: the number of $b$-tagged jets and the transverse momentum of the sixth leading jet, $p_{\mathrm {T}}^{\mathrm {6th\ jet}}$. The correlation in $t\bar{t}$ events is estimated in simulation to be $\rho =0.009$. The six regions, defined by three bins of the number of $b$-tagged jets and two ranges in $p_{\mathrm {T}}^{\mathrm {6th\ jet}}$, are detailed in Table 2. Region $F$, which is the signal region, i.e. two $b$-tagged jets with $p_{\mathrm {T}}^{\mathrm {6th\ jet}}>30\mathrm{\,GeV}$, contains the largest fraction of $t\bar{t}$ events in addition to multijet background events.

**Table 2:** Event yields for the six regions, defined by the number of $b$-tagged jets and the transverse momentum of the sixth leading jet $p_{\mathrm {T}}^{\mathrm {6th\ jet}}$, are listed for data and $t\bar{t}$ simulation assuming $m_{t}=172.5\mathrm{\,GeV}$ with statistical uncertainty. The $t\bar{t}$ fractions are derived from the observed numbers of events and their statistical uncertainties

	$p_{\mathrm {T}}^{\mathrm {6th\ jet}}\le 30\mathrm{\,GeV}$			$p_{\mathrm {T}}^{\mathrm {6th\ jet}}> 30\mathrm{\,GeV}$		
		Data events $N_R^{\mathrm {obs}}$	Signal MC events $N_R^{\mathrm {sig}}$		Data events $N_R^{\mathrm {obs}}$	Signal MC events $N_R^{\mathrm {sig}}$
$b$-Tagged jets	Region $R$	signal fraction		Region $R$	signal fraction	
$0$	$A$	$93,732$	$306\pm 4$	$B$	$286,416$	$2607\pm 11$
		$0.33\pm 0.01\,\%$			$0.91\pm 0.01\,\%$	
$$1$$	$$C$$	$$23,536$$	$$ 678\pm 5$$	$$D$$	$$ 77,301$$	$$5117\pm 14$$
		$2.88\pm 0.04\,\%$			$6.62\pm 0.04\,\%$	
$$2$$	$$E$$	$$ 4,532$$	$$ 399\pm 5$$	$$F$$	$$ 15,551$$	$$2582\pm 13$$
		$8.80\pm 0.29\,\%$			$16.60\pm 0.27\,\%$	

**Fig. 2:**
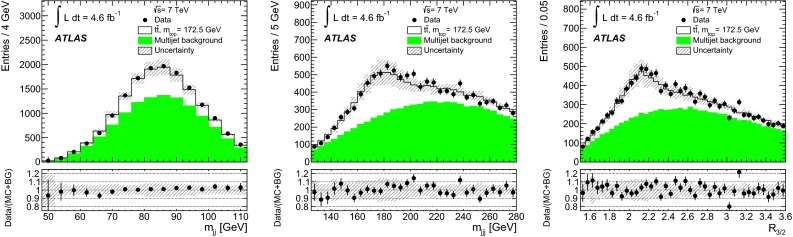
Distributions of (*left*) dijet mass $m_{jj}$, (*middle*) three-jet mass $m_{jjj}$, and (*right*) ratio of three-jet mass to dijet mass $R_{3/2}$, measured in data and compared to expectations after applying all analysis event selection criteria (i.e. for region $F$). The shape and normalisation of the multijet background distributions (*green shaded histograms*) are calculated using Eq. (4). The distributions for the $t\bar{t}$ events (*white histograms*) are taken from the MC simulation using a top-quark mass value of $172.5\mathrm{\,GeV}$. The *insets* under the distributions show the ratio of data to the summed contributions of $t\bar{t}$ MC signal and modelled multijet background (see Sect. 4). The *error bars* represent the statistical uncertainties on the data. The *shaded bands* show the statistical and systematic (see Sect. 6) uncertainty on the expected signal and background distributions

Regions $A$ through $E$ are depleted in $t\bar{t}$ events, but enhanced in multijet background events. The data yields in these regions ($N_R^{\mathrm {obs}}$, $R=A, \ldots , E$) and the expected number of $t\bar{t}$ events from MC simulation, $N_R^{\mathrm {sig}}$, using $m_t=172.5\mathrm{\,GeV}$ are listed in Table 2. The table also quotes the derived fraction $N_R^{\mathrm {sig}}/N_R^{\mathrm {obs}}$ of $t\bar{t}$ events in the respective region. The $t\bar{t}$ event fraction in each region other than $F$ is accounted for by subtracting from data, $N_R^{\mathrm {obs}}$, the number of $t\bar{t}$ events predicted by the MC simulation, $N_R^{\mathrm {sig}}$, for a top-quark mass value of $175\mathrm{\,GeV}$:

2 $$\begin{aligned} N_R^{\mathrm {bkg}} = N_R^{\mathrm {obs}} - N_R^{\mathrm {sig}} \end{aligned}$$

for region $R=A, \ldots , E$. Due to the small $t\bar{t}$ fractions in region $A$ to $E$, the top-quark mass value chosen in the simulation used for this subtraction procedure marginally affects the value of $m_{t}$ measured in this analysis. Therefore, the value of $m_{t}$ closest to the measured value (see Sect. 5) is used in the simulation for subtraction. The small dependence on the $t\bar{t}$ MC simulation introduced by this subtraction is accounted for by a systematic uncertainty (see Sect. 6.2).

Given the tiny correlation of $0.9\,\%$ predicted by MC simulation studies for the two observables used to define the regions, the total number of multijet background events, $N_F^{\mathrm {bkg}}$, in region $F$ can be estimated by cross-multiplication, for example, from the ratio of the number of events in region $B$ to region $A$ scaled by the number of events in region $E$. To obtain the distribution of multijet background events, $N_F^{\mathrm {bkg}}(x)$, for any given observable $x$ (e.g. $R_{3/2}$) to the distribution in region $F$ either of the following formulae can be used:

3 $$\begin{aligned} N_F^{\mathrm {bkg}}(x) = N_E^{\mathrm {bkg}}\cdot \frac{N_B^{\mathrm {bkg}}(x)}{N_A^{\mathrm {bkg}}}\qquad {\mathrm {or}} \nonumber \\ N_F^{\mathrm {bkg}}(x) = N_E^{\mathrm {bkg}}\cdot \frac{N_D^{\mathrm {bkg}}(x)}{N_C^{\mathrm {bkg}}} ,{} \end{aligned}$$

hence

4 $$\begin{aligned} N_F^{\mathrm {bkg}}(x)=\frac{N_E^{\mathrm {bkg}}}{2} \cdot \left( \frac{N_B^{\mathrm {bkg}}(x)}{N_A^{\mathrm {bkg}}}+\frac{N_D^{\mathrm {bkg}}(x)}{N_C^{\mathrm {bkg}}}\right) . \end{aligned}$$

Here, $N_B^{\mathrm {bkg}}(x)$ and $N_D^{\mathrm {bkg}}(x)$ define the shape of the distributions for an observable $x$, while the appropriate normalisation is achieved by scaling with the total number of events ($N_A^{\mathrm {bkg}}$, $N_C^{\mathrm {bkg}}$, $N_E^{\mathrm {bkg}}$) in the respective region. Equation (4) is used to determine the multijet background while Eqs. (3) are used to estimate the systematic uncertainties on the modelled background (see Sect. 6.2).

Figure 2 shows the distributions of the dijet mass, the three-jet mass, and their ratio, $R_{3/2}=m_{jjj}/m_{jj}$, after applying the event selection and jet assignments detailed in Sect. 3. In calculating $R_{3/2}$ values for an event, $m_{jjj}$ of both top-quark candidates and $m_{jj}$ of the related $W$ boson candidate are considered. Superimposed in Fig. 2 is the sum of the distributions for the $t\bar{t}$ events obtained from MC simulation using $m_t=172.5\mathrm{\,GeV}$ plus the multijet background estimated using Eq. (4). The distributions of the ratios of data to the sum of the signal MC events plus background model seen in Fig. 2 show that the data-driven approach yields a reliable model of the multijet background.

## Top-quark mass measurement

The top-quark mass is obtained from a binned likelihood fit to the $R_{3/2}$ distribution shown in Fig. 2. As noted above, two values of $R_{3/2}$ are contributed by each event, reconstructed separately from the top and antitop-quark candidates. Because equal masses are assumed for the Breit–Wigner lineshapes for the top quark and antiquark in the kinematic fit for the jet assignments, the two values are correlated at the level of approximately 60 % according to MC simulation. This is corrected for in the statistical treatment described below. Templates are created for both the simulated top-quark contribution to the $R_{3/2}$ distribution and the modelled background distribution. The top-quark contribution is parameterised by the sum of a Gaussian function and a Landau function which account, respectively, for the correctly reconstructed top-quark events and for the combinatorial background due to mis-assignment of jets to partons (see Sect. 3). This description involves six parameters.

**Fig. 3:**
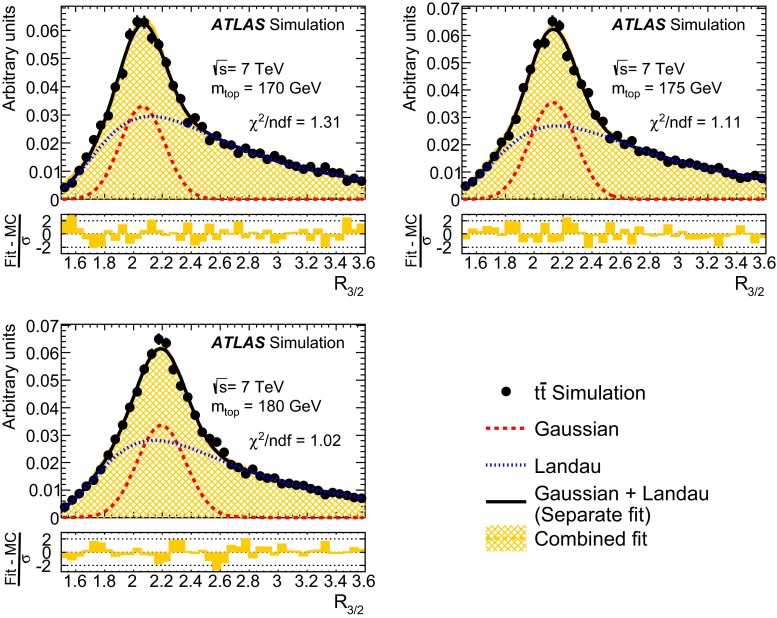
Templates for the $R_{3/2}$ distribution for $t\bar{t}$ MC simulation using top-quark mass values of $170.0,\ 175.0$ and $180.0\mathrm{\,GeV}$, respectively. For each top-quark mass, the $R_{3/2}$ distribution is fitted by the sum (*black solid*) of a Gaussian (*red dashed*) and Landau (*blue dotted*) function. Superimposed (*orange cross-hatched*) are the templates obtained from a combined fit of all $R_{3/2}$ distributions using a linear dependence of parameters of the Gaussian and Landau functions on the top-quark mass value. The *insets* under the distributions show the difference Fit$-$MC between the combined fit and the simulated $R_{3/2}$ histogram normalised to the statistical uncertainty $\sigma$ of the corresponding $R_{3/2}$ bin

A two-step approach is used to obtain an $m_{t}$-dependent representation of the templates. Firstly, the $R_{3/2}$ distribution from each of the seven simulation samples of different $m_{t}$ is fitted separately to determine the six parameters for each template mass. This yields a good description of the $R_{3/2}$ distributions per chosen $m_t$ (see Fig. 3). MC simulation has shown that each of the six parameters of the Gaussian and Landau functions depend linearly on the input top-quark mass. Secondly, from the parameter values obtained by these separate fits, initial values for offsets and slopes of the linear $m_t$ dependencies are derived and then used as inputs to a combined, simultaneous fit to all seven $R_{3/2}$ distributions. In total 12 parameters are determined by the combined fit, which yields a $\chi ^2$ per number of degrees of freedom (ndf) of $\chi ^2/\mathrm {ndf}=298/282=1.06$. Both the individual and the combined fit results are shown for three of the seven $m_t$ values in Fig. 3.

**Fig. 4:**
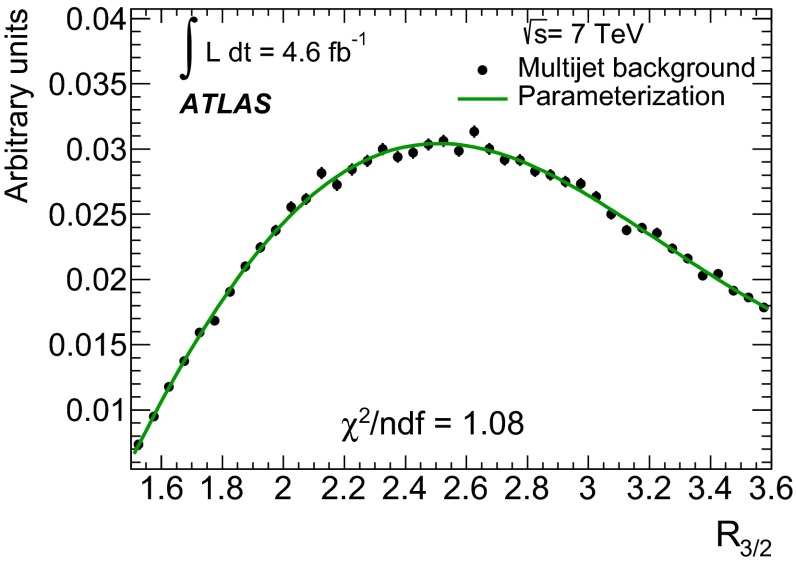
Distribution of $R_{3/2}$ for multijet background events according to the data-driven prescription of Eq. (4), normalised to unit integral. The parameterisation of the distribution by the sum of a Gaussian function and a linear function is superimposed

The modelled multijet background, obtained using Eq. (4), is parameterised by a Gaussian function plus a linear function, thus involving five parameters. The resulting fit to data is shown in Fig. 4 and yields $\chi ^2/$ndf$=40/36=1.08$. The shape of the fitted parameterisation is assumed to be independent of the top-quark mass while the normalisation is obtained from fitting to the data distribution. Any residual dependence of this parameterisation on the top-quark mass is accounted for by a systematic uncertainty (see Sect. 6).

The $R_{3/2}$ distribution is fitted for the top-quark mass using the templates for both the top-quark signal and the modelled multijet background distribution described above. Defining the likelihood function as a product of Poisson probabilities

5 $$\begin{aligned} \mathcal {L}(R_{3/2}|m_{t})= \prod _{j}^{\mathrm {bins}} \left( \frac{\lambda _{j}^{N_{F,j}^{\mathrm {obs}}}}{N_{F,j}^{\mathrm {obs}}!} \right) \exp ({-\lambda _{j}}), \end{aligned}$$

a binned likelihood fit is applied. For the $R_{3/2, j}$, i.e. the $j^{\mathrm {th}}$ bin of the $R_{3/2}$ distribution, $N_{F,j}^{\mathrm {obs}}\equiv N_F^{\mathrm {obs}}(R_{3/2, j})$ and $\lambda _{j}$ are the observed and expected number of events in that bin. Here, the expected number of events in a bin is given by the sum of $t\bar{t}$ events $N_{F,j}^{\mathrm {sig}}(m_{t})$, as derived from the signal templates, and multijet background events $N_{F,j}^{\mathrm {bkg}}\equiv N_F^{\mathrm {bkg}}(R_{3/2, j})$,

6 $$\begin{aligned} \lambda _{j} = (1-f_{\mathrm {bkg}}) N_{F,j}^{\mathrm {sig}}(m_{t}) + f_{\mathrm {bkg}} N_{F,j}^{\mathrm {bkg}}, \end{aligned}$$

where $f_{\mathrm {bkg}}$ is the fraction of multijet background events, which is determined by the fit.

**Fig. 5:**
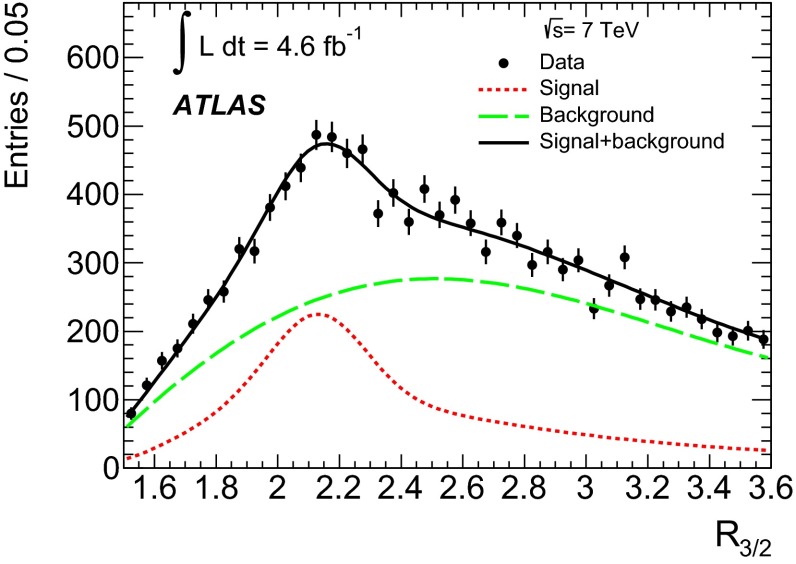
Result of the fit of Eq. (5) (solid black) to the measured $R_{3/2}$ distribution. The red dotted curve shows the contribution from top-quark events and corresponds to the black curve in Fig. 3; the green dashed line is the modelled multijet background

Equation (5) is maximised with respect to $m_{t}$ and $f_{\mathrm {bkg}}$ for $R_{3/2}$ values between $1.5$ and $3.6$, taking the normalisation from data, yielding

7 $$\begin{aligned} m_{t} = 175.06 \pm 1.35\;\text{(stat.) }\mathrm{\,GeV}\end{aligned}$$

for a background fraction of $f_{\mathrm {bkg}}=0.72 \pm 0.01$ and $\chi ^2/$ndf $=48/39=1.23$. The difference between the fitted background fraction and the value quoted in Sect. 3.3 is due to the restricted $R_{3/2}$ range used in the fit. The result of this fit is shown in Fig. 5. The $\chi ^2/$ndf value is enlarged by the statistical correlation between the two $R_{3/2}$ values from each event. Its impact has been incorporated in the quoted statistical uncertainty^3^ of Eq. (7) as follows.

**Fig. 6:**
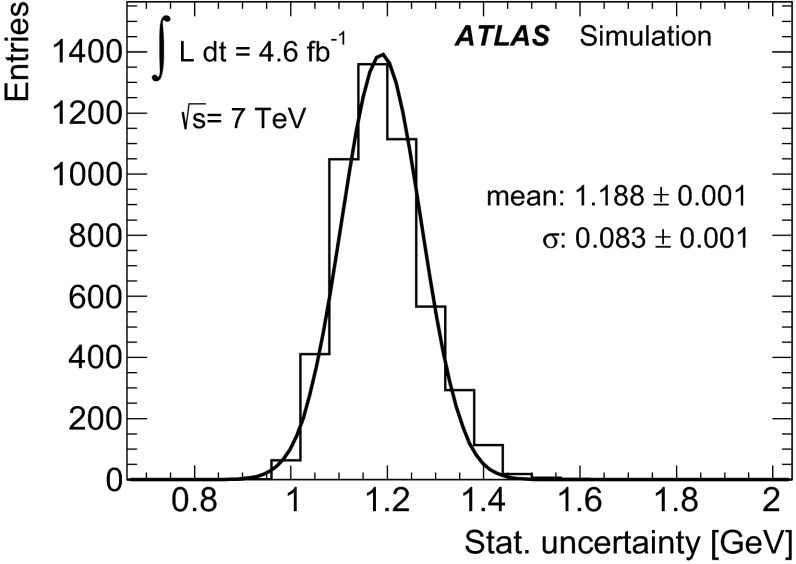
Expected statistical uncertainty on the top-quark mass obtained from 5000 pseudo-experiments using $t\bar{t}$ MC simulation events assuming $m_{t}=175\mathrm{\,GeV}$ and neglecting correlations between the two $R_{3/2}$ values per event

The statistical uncertainty of the fit is studied by performing pseudo-experiments, where 5000 pseudo-datasets of $R_{3/2}$ values, each statistically equivalent to the data, are assembled from values randomly picked from signal and background histograms^4^. They are obtained from $t\bar{t}$ MC simulation^5^ generated for $m_{t}=175\mathrm{\,GeV}$, and from the multijet background estimate, detailed in Sect. 4, respectively. Pseudo-datasets are created from two-dimensional histograms for the full MC sample of $R_{3/2}$ from the top-quark candidate versus $R_{3/2}$ of the top-antiquark candidate in an event, thereby accounting for the $60\,\%$ correlation. Similarly, one-dimensional histograms are used to produce pseudo-datasets which do not include the correlations. The top quark mass and its statistical uncertainty are evaluated for each pseudo-dataset, using the likelihood fit of Eq. (5)

The expected statistical uncertainty of the fit when neglecting the correlation is shown in Fig. 6. A fit of a Gaussian function to the output of the 5000 pseudo-experiments yields an expected statistical uncertainty of $1.19\pm 0.08\mathrm{\,GeV}$, which agrees with the observed statistical uncertainty of $1.15\mathrm{\,GeV}$.

The same procedure with 5000 pseudo-datasets is applied to each of the seven top-quark mass values used for MC simulation, considering the correlation of the $R_{3/2}$ values for the top quark and antiquark candidates in an event. Distributions of the pull values for the 5000 pseudo-datasets are derived, where the pull is the difference between the fitted, $m_{t}^{\mathrm {fit}}$, and input, $m_{t}^{\mathrm {inp}}$, top-quark mass values divided by the statistical uncertainty, $\sigma ^{\mathrm {fit}}$, of the fit; $\mathrm {pull}=(m_{t}^{\mathrm {fit}}-m_{t}^{\mathrm {inp}})/\sigma ^{\mathrm {fit}}$. The pull distribution for an unbiased measurement has a mean of zero and a standard deviation of unity. For this measurement no dependence of the pull mean on $m_{t}^{\mathrm {inp}}$ is observed. An average pull mean value corresponding to $m_{t}^{\mathrm {fit}}-m_{t}^{\mathrm {inp}} = -0.23 \pm 0.14\mathrm{\,GeV}$ and an average pull width of $1.175 \pm 0.027$ are obtained. The bias in the width of the pull is due to the statistical correlation. To correct for this bias, the observed statistical uncertainty of $1.15\mathrm{\,GeV}$ is scaled by 1.175 to yield the statistical uncertainty of $1.35\mathrm{\,GeV}$ quoted in Eq. (7). The bias indicated by the non-zero mean value of the pull distribution is corrected for in the above quoted result. The uncertainty of the pull mean value is considered as part of the systematic uncertainty related to the calibration of this measurement method.

## Systematic uncertainties

A large number of potential sources of systematic uncertainty were evaluated. They can be categorised as uncertainties due to: (i) the modelling of the $t\bar{t}$ events in the MC simulation, (ii) the modelling of the multijet background by the data-driven approach, (iii) the correction and calibration of the energies of the reconstructed jets, the jet reconstruction and the $b$-quark identification efficiency. These are described in detail in Sects. 6.1–6.3. In general, for every investigated source of systematic uncertainty the likelihood fit of Eq. (5) for the top-quark mass is repeated with a modified parameter. Any change of the measured top-quark mass is assigned as the systematic uncertainty due to this source. The total systematic uncertainty arises from adding all individual contributions in quadrature. Table 3 lists the individual contributions and their combination. The largest systematic uncertainties are due to the jet and $b$-jet energy scales and the hadronisation modelling.

### Signal modelling

All systematic uncertainties related to the modelling of $t\bar{t}$ events and the lineshape of the top-quark mass distribution are investigated using 5000 data sets, created by the resampling technique described in Sect. 5 by randomly selecting $R_{3/2}$ values from a distribution of $t\bar{t}$ MC simulation events generated with a shifted value for the relevant parameter as detailed below. In Table 3, the difference between the mean values obtained with shifted and with default parameter values, from 5000 pseudo-experiments each, is quoted for the investigated sources of systematic uncertainty.

**Table 3:** Compilation of investigated systematic uncertainties on the determined top-quark mass reported in Sect. 5. The three parts of the table correspond to uncertainties in the $t\bar{t}$ and multijet background modelling, and uncertainties in the jet measurements

Signal modelling:	$\Delta m_{t}$ ( GeV)
Method calibration	0.42
Trigger	0.01
Signal MC generator	0.30
Hadronisation	0.50
Fast simulation	0.24
Colour reconnection	0.22
Underlying event	0.08
ISR and FSR	0.22
Proton PDF	0.09
Pile-up	0.02
Background modelling:	$\Delta m_{t}$ ( GeV)
Multijet background	0.35
Jet measurements:	$\Delta m_{t}$ ( GeV)
Jet energy scale (see Table 4)	0.51
$b$-jet energy scale	0.62
Jet energy resolution	0.01
Jet reconstruction efficiency	0.01
$b$-tag efficiency and mistag rate	0.17
Soft contributions to missing energy	0.02
JVF scale factors	0.02
Total systematic uncertainty	1.22

**Method calibration** Our particular choice of signal parameterisation functions and the adopted linear dependence of the parameters of these functions on the top-quark mass value can affect the reconstructed top-quark mass. This uncertainty is estimated from the differences between the fitted and the input top-quark mass value when determining the $t\bar{t}$ template for each of the seven simulation samples separately. The average of the absolute differences is $0.23\mathrm{\,GeV}$ and also accounts for the average shift of the pull distributions.

The shapes of the templates for $t\bar{t}$ and multijet background events can be affected by statistical uncertainties of either simulated events (signal templates) or data (background templates). This is assessed by creating 1000 new sets of templates by letting the standard templates fluctuate within their statistical uncertainties. The top-quark mass values obtained with these new templates are found to have an RMS spread of $0.42\mathrm{\,GeV}$.

The larger of 0.23 and $0.42\mathrm{\,GeV}$ is assigned as a systematic uncertainty for the method calibration.

**Trigger** Studies of the trigger efficiency close to the threshold region reveal a 5 % difference between data and MC simulation. The impact of this deviation is evaluated by reweighting the efficiency for triggering MC simulation events to match the efficiency observed in data as a function of the transverse momentum of the fifth leading jet. The observed change in the measured top-quark mass is $0.01\mathrm{\,GeV}$.

**Signal MC generator** The impact of the choice of Powheg-box as the signal MC generator is evaluated by generating $$t\bar{t}$$ events at $$m_{t}=$$$$172.5\mathrm{\,GeV}$$ using either Powheg-box or MC@NLO [44, 45], each with Herwig [46] for the modelling of the parton shower and the hadronisation. The full difference in the top-quark mass values of $$0.30\mathrm{\,GeV}$$ found from using Powheg or MC@NLO to determine the signal templates is quoted as the systematic uncertainty.

**Hadronisation** Potential systematic uncertainties due to our choice of parton shower and hadronisation model are assessed by using Powheg$t\bar{t}$ events with parton shower and hadronisation performed by either Pythia with the Perugia P2012 tune or by Herwig^6^ and Jimmy with the ATLAS AUET2 tune [47]. The full difference in the top-quark mass values of $0.50\mathrm{\,GeV}$ between these two samples is ascribed to the uncertainty due to parton shower and hadronisation modelling.

**Fast simulation** The $t\bar{t}$ MC simulation events for all seven $m_{t}$ mass values are processed by a fast simulation of the ATLAS detector [22, 48]. For $m_{t}=172.5\mathrm{\,GeV}$ an additional $t\bar{t}$ MC simulation sample is created using the full simulation of the ATLAS detector. The systematic uncertainty of $0.24\mathrm{\,GeV}$ is estimated from the difference of $0.24\pm 0.30\;\text{(stat.) }\mathrm{\,GeV}$ between the top-quark masses obtained by performing pseudo-experiments on either the fast or the full MC simulation sample.

**Colour reconnection** Consequences of reconnection of colour flux lines between the partons are estimated with Powheg-box and Pythia by comparing simulated $t\bar{t}$ events based on the Perugia 2012 tune including colour reconnection (CR) and the Perugia 2012loCR tune [29], which uses a lower colour reconnection strength than the default tune. The full difference of $0.22\mathrm{\,GeV}$ in measured top-quark mass between these two samples is attributed to the uncertainty from colour reconnection.

**Underlying event** The potential uncertainty due to the choice of a particular model to simulate underlying events is evaluated by considering events simulated using Powheg-box and Pythia based on the Perugia 2012 tune and comparing to events based on the Perugia 2012mpiHi tune [29], which has an increased rate of jets from multi-parton interactions. Both tunes use the same parameters for the modelling of colour reconnection and both predict similar activity in the plane transverse to the leading charged particle. The samples used for colour reconnection uncertainties are based on different values for these parameters. The full difference between the fitted mass values of $0.08\mathrm{\,GeV}$ is taken as the systematic uncertainty.

**Initial- and final-state QCD radiation** The impact from additional jets due to initial- and final-state QCD radiation, ISR and FSR, respectively, on the top-quark mass measurement is analysed with dedicated $t\bar{t}$ event samples generated with the leading-order generator AcerMC [49]. Parton showering and hadronisation are performed by Pythia using the Perugia 2011C tune. Tunable parameters that control the parton shower strength are varied up and down in these samples in a range for which the simulated radiation in $t\bar{t}$ events is compatible with the results found from an investigation of additional jets in $t\bar{t}$ events [50]. Half of the full difference between the measured top-quark masses from these two samples is taken as the systematic uncertainty, which is $0.22\mathrm{\,GeV}$.

**Proton–parton distribution function** The $t\bar{t}$ event samples were generated using CT10 PDF. The uncertainties in these PDFs are specified by 26 pairs of additional PDF sets provided by the CTEQ group [51]. The effect of the PDF uncertainties on the $t\bar{t}$ templates is derived from samples generated using MC@NLO with Herwig for hadronisation. For every additional PDF set, the simulated events are reweighted by the ratio of the varied PDF to the central PDF. Signal templates are constructed for each of these 26 pairs of sets. Using these templates, pseudo-experiments are performed per pair of PDF sets but using the same events for the up and down variations within every pair to alleviate the effects of the statistical fluctuations. Half of the sum in quadrature of the difference within each of the 26 pairs is assigned as the systematic uncertainty derived from the CTEQ PDF. Additionally, the $t\bar{t}$ event samples are also reweighted to the central PDF set of either MSTW2008 [52] or NNPDF23 [53]. The final systematic uncertainty due to PDF is the sum in quadrature of these three contributions, which yields $0.09\mathrm{\,GeV}$.

**Pile-up** The consequences of additional proton–proton interactions on the top-quark mass measurement are investigated by repeating the full analysis separately as a function of the number of reconstructed collision vertices, $n_{\mathrm {vtx}}$, and as a function of the average number, $\langle \mu \rangle$, of inelastic proton–proton interactions per bunch crossing. This is in addition to the effects already accounted for in the corresponding jet energy scale. The data sample is split into disjoint subsamples of $n_{\mathrm {vtx}}\le 5$, $5<n_{\mathrm {vtx}}\le 7$, and $7<n_{\mathrm {vtx}}$, or into subsamples of $\langle \mu \rangle \le 6$, $6<\langle \mu \rangle \le 10$, and $10<\langle \mu \rangle$. In each of these subsamples the full analysis for the top-quark mass measurement is repeated, giving per-subsample variations, $\Delta m_{t}$. Within large statistical uncertainties, data and MC simulation agree. The effect of any residual differences between data and simulation is included by scaling $\Delta m_{t}$ with the absolute difference between the $n_{\mathrm {vtx}}$ distribution in data and simulation, each normalised to unit integral. The scaled $\Delta m_{t}$ obtained for each of the three subsamples are summed, yielding $0.02\mathrm{\,GeV}$. The same procedure is applied to the $\Delta m_{t}$ from the subsamples of the $\langle \mu \rangle$ distribution, yielding $0.01\mathrm{\,GeV}$. The two sums, derived from the $n_{\mathrm {vtx}}$ and for $\langle \mu \rangle$ distributions, are then added in quadrature to estimate the systematic uncertainty on the top-quark mass measurement of $0.02\mathrm{\,GeV}$.

### Background modelling

Each of the prescriptions in Eq. (3) yields an independent estimate of the multijet background to the $$t\bar{t}$$ events. Employing these separately distinguishes different contributions from background processes and accounts for conceivable correlations between the distribution $$N_F^{\mathrm {bkg}}(x)$$ and the multiplicity of the $$b$$-tagged jets. In particular, the regions $$C$$ and $$D$$, where one jet is $$b$$-tagged, accumulate background from single top-quark production while suppressing contributions from $$W$$$$+$$ jets processes. The regions $$A$$ and $$B$$, where no jets are $$b$$-tagged, are essentially free from $$t\bar{t}$$ events and, hence, insensitive to systematic uncertainties from the subtraction of residual $$t\bar{t}$$ contributions (see Eq. (2)). The average of the absolute shifts on $$m_{t}$$ when using either of the prescriptions in Eq. (3) separately is taken as symmetric uncertainty on the background modelling, which amounts to $$0.35\mathrm{\,GeV}$$.

### Jet measurement

Systematic uncertainties due to measuring jets are listed in Table 3 and detailed in the following.

**Jet energy scale** The relative jet energy scale uncertainty varies between about 1 and $3\,\%$ depending on the $p_{\mathrm {T}}$ and $\eta$ of the jet. This was investigated in detail in Refs. [17, 34, 35], which prescribe 21 components of uncertainty, including a proper treatment of the correlations between the individual sources. The 21 components involve nuisance parameters from different in situ techniques applied to evaluate residual jet energy scale correction factors which account for differences between data and MC simulation. They originate from the calibration method, the calorimeter response, the detector simulation and the specific choice of parameters in the physics model employed by the MC event generator. Further sources of uncertainty are related to the extrapolation to the high-$p_{\mathrm {T}}$ region, to the intercalibration of jets at large pseudorapidity with central jets and to the pile-up. Topology-dependent uncertainties arising from the relative numbers of jets initiated by gluons and light quarks are included as well as uncertainties on the response to jets with nearby hadronic activity. The 21 components are considered uncorrelated. After repeating the top-quark mass measurement separately for each component, the variation in the top-quark mass value obtained from the up and down variation of each nuisance parameter is symmetrised. The individual symmetrised contributions are added in quadrature to estimate the overall $\Delta m_{t}$ due to jet energy scale uncertainty of $0.51\mathrm{\,GeV}$.

Table 4 lists the individual systematic uncertainty components related to the energy measurements of jets combined into different categories according to the type of source and correlations (see Ref. [34]).

**Table 4:** Individual contributions to the systematic uncertainty of the top-quark mass due to uncertainties on the jet energy scale listed in Table 3

	$$\Delta m_{t}$$ ( GeV)
Statistics and method	0.09
Physics modelling	0.31
Detector description	0.36
Mixed detector and modelling	0.05
Single high-$p_{\mathrm {T}}$ particle	0.02
Relative non-closure in MC	0.04
Pile-up	0.03
Close-by jets	0.02
Flavour composition and response	0.10
Jet energy scale	0.51
$b$-jet energy scale	0.62

**Relative**$b$**-jet energy scale** The relative $b$-jet energy scale accounts for the remaining differences between an inclusive jets sample and jets originating from bottom quarks after the global jet energy scale is determined. It is estimated by choosing different fragmentation models. An extra uncertainty, ranging between $1.8$ and $0.7\,\%$, and decreasing as jet $p_{\mathrm {T}}$ increases, is assigned to each $b$-jet to account for the difference between jets containing $b$-flavoured hadrons and the inclusive jet sample. This uncertainty is derived from MC simulation studies and validated by comparison with data (see Ref. [36] for details). For the spectrum of jets selected in this analysis the average uncertainty is less than $1.2\,\%$. The systematic uncertainty on $m_t$ due to the relative $b$-jet energy scale is $0.62\mathrm{\,GeV}$.

**Jet energy resolution** The impact of a residual difference between the jet energy resolution in data and MC simulation is accounted for by smearing the energy of each reconstructed jet in the simulation by a Gaussian function before applying the event selection requirements (see Ref. [54] for details). The top-quark mass measurement is repeated using the smeared jet energies yielding a variation of $0.01\mathrm{\,GeV}$, which is symmetrised and assigned as a systematic uncertainty.

**Jet reconstruction efficiency** The jet reconstruction efficiency was found in Ref. [17] to differ in data and MC simulation by no more than $\pm 2\,\%$. This residual difference is applied as a variation by randomly removing jets from the simulated events before applying the event selection criteria. The variation of $0.01\mathrm{\,GeV}$ found by repeating the top-quark mass measurement employing this modified MC simulation sample is taken as a systematic uncertainty.

$b$**-tagging efficiency and mistag rate** The efficiency for tagging $b$-quark jets as well as the $c$-quark and light-quark ($u$, $d$, $s$) jet mistag rate in simulation are corrected to data by scale factors [19, 39]. The uncertainty of this correction is propagated to the measured top-quark mass by varying these scale factors by one standard deviation about their central values, which depend on the $p_{\mathrm {T}}$ and the $\eta$ of the jet, and on the underlying quark flavour. The variations in the top-quark mass are added in quadrature to assess the systematic uncertainty from this source, which yields $0.17\mathrm{\,GeV}$.

**Soft contribution to missing energy** Measured energy deposits in the calorimeter which are not associated with a high-$p_{\mathrm {T}}$ jet, photon, electron, or muon, stem mostly from low-$p_{\mathrm {T}}$ particles. These energy deposits are calibrated using the local hadronic calibration scheme [40]. An uncertainty of $0.02\mathrm{\,GeV}$ on the top-quark mass due to this assumption is derived by scaling the soft contributions within their uncertainties.

**Jet vertex fraction scale factor uncertainty** The difference in JVF between data and MC simulation is corrected by applying scale factors. These scale factors, varied according to their uncertainty, are applied to MC simulation events as a function of the $p_{\mathrm {T}}$ of a jet. The resulting variation in the measured top-quark mass amounts to $0.02\mathrm{\,GeV}$.

## Comparison with alternative analysis

The result of this measurement is compared with an independent measurement based on essentially the same selection described in Sect. 3. For this independent measurement, however, entirely different methods are chosen for alleviating the effects due to uncertainties from the jet energy measurement and for modelling the multijet background. Applying a simultaneous two-dimensional fit to the $W$ boson and top-quark masses unfolds the dependency of the top-quark mass on a global jet scale factor. Thus systematic uncertainties affecting the jet scale factor are mostly removed from the uncertainties in the measured top-quark mass; however, this gives rise to increased statistical uncertainty (see also Ref. [15]).

In the independent alternative measurement, the multijet background is modelled using an event mixing procedure. Here, events with six or more jets are composed from events with exactly five jets, two of which are $b$-tagged, merged with the sixth and subsequent leading jets from events of an independent inclusive jet sample. Kinematic similarity of the two events to be mixed is ensured by requiring the similarity of the transverse momenta of both the leading jets in the two events and also of the fifth leading jets. Evaluation of the systematic uncertainties described in Sect. 6 was performed for this independent analysis. This investigation showed that the alternative analysis and the main analysis have similar sensitivities to the top-quark mass. The alternative analysis has yielded a top-quark mass value and a total statistical uncertainty of $m_{t} = 174.7 \pm 1.4\ (\mathrm {stat.+JSF}) \mathrm{\,GeV}$ with a global jet scale factor of $\mathrm {JSF} = 1.013 \pm 0.008\, (\mathrm {stat.})$, in good agreement with the results presented in Sects. 5 and 6.

## Summary

In a data set corresponding to $4.6\,\text{ fb }^{-1}$ of proton–proton collisions collected by the ATLAS experiment at the LHC at $\sqrt{s}=7\mathrm{\,TeV}$, events consistent with $t\bar{t}$ pairs decaying into a fully hadronic final state were selected. A kinematic likelihood fit was employed to assign reconstructed jets to the partons expected from the leading-order hadronic decay of the intermediate $t\bar{t}$ state. To reduce the sensitivity of the analysis to the energy scale of jets, the ratio $R_{3/2}$ of the three-jet mass to the dijet mass was calculated. The three-jet mass calculation combines all jets from a top-quark decay, and the dijet mass is computed with the two jets from the hadronically decaying $W$ boson. The multijet background was determined by dividing the event sample into six disjoint sets according to the number of $b$-tagged jets and the $p_{\mathrm {T}}$ of the sixth jet. The background in the region of interest is then estimated by cross-multiplication. Fitting the $R_{3/2}$ distribution for the top-quark mass yields

8 $$\begin{aligned} m_{t} = 175.1 \pm 1.4\;\text{(stat.) }\pm 1.2\;\text{(syst.) }\mathrm{\,GeV}\end{aligned}$$

with a measured fraction of background events $f_{\mathrm {bkg}}=0.72\pm 0.01$. The systematic uncertainties are dominated by the residual uncertainties from the jet energy scale for all jets and, specifically, for $b$-quark jets and by the uncertainties from hadronisation modelling. The total uncertainty is $1.8\mathrm{\,GeV}$. This result has a precision similar to, and within uncertainties fully agrees with, the top-quark mass measured from the fully hadronic final state by other experiments [10, 11] and the result measured in the lepton plus jets final state and published previously by ATLAS [15].

## Footnotes

1 ATLAS uses a right-handed coordinate system with its origin at the nominal interaction point (IP) in the centre of the detector and the $z$-axis along the beam pipe. The $x$-axis points from the IP to the centre of the LHC ring, and the $y$ axis points upward. Cylindrical coordinates $(r,\phi )$ are used in the transverse plane, $\phi$ being the azimuthal angle around the beam pipe. The pseudorapidity is defined in terms of the polar angle $\theta$ as $\eta =-\ln \tan (\theta /2)$. The transverse momentum $p_{\mathrm {T}}$ lies in the $x$–$y$ plane.

2 Distances between particles or jets are measured using $\Delta R = \sqrt{(\Delta \phi )^2 + (\Delta \eta )^2}$ where $\Delta \phi$ and $\Delta \eta$ are the differences in $\phi$ and $\eta$ between the two objects.

3 The uncorrected statistical uncertainty obtained from the fit yields 1.15 GeV.

4 The signal histograms used to draw pseudodata include $\approx$ 45,000 events for the 172.5 GeV mass point sample and 4500–6500 events for the remaining mass points. The background histogram is derived using $\approx$ 230,000 data events in the control regions defined in Sect. 4.

5 A single event may be used several times in different data sets. The correlation introduced by this resampling technique is corrected in all distributions and results presented in this paper as described in Ref. [43].

6 Version 6.520 of Herwig was used with default parameters (expect for clpow$=1.2$).
